# Association between anatomical subtypes of medullary infarction and clinical outcome: a multicenter cohort study

**DOI:** 10.3389/fneur.2026.1770888

**Published:** 2026-03-24

**Authors:** Linhu Zhao, Yu Gu, Hongli Liu, Gang Tang, Chunyan Lei, Ansong Jin, Qionghua Deng, Ruolong Xue, Xinglong Yang, Xiaoyan Zhu

**Affiliations:** 1 First Department of Neurology, First Affiliated Hospital of Kunming Medical University, Kunming, Yunnan, China; 2 Department of Neurology, Peking University First Hospital Taiyuan Hospital, Taiyuan, Shanxi, China; 3 West China Hospital, Sichuan University, Chengdu, Sichuan, China; 4 Second Department of Neurology, First Affiliated Hospital of Kunming Medical University, Kunming, Yunnan, China

**Keywords:** anatomy, lateral medullary infarction, medial medullary infarction, medulla oblongata, prognosis

## Abstract

**Objective:**

This multicenter, large-sample retrospective cohort study aimed to investigate the clinical characteristics, early neurological deterioration (END), clinical outcome, and prognostic factors among seven anatomical subtypes of acute medullary infarction, and to examine whether anatomical classification provided independent prognostic value beyond baseline neurological severity.

**Methods:**

From January 2019 to October 2024, 352 patients with acute medullary infarction from three centers (the First Affiliated Hospital of Kunming Medical University, West China Hospital of Sichuan University, and Peking University First Hospital Taiyuan Hospital) were enrolled. Based on axial magnetic resonance imaging—diffusion weighted imaging findings, patients were classified into seven anatomical subtypes: lateral medullary infarction (LMI) subtypes (superficial lateral, dorsolateral, oblique lateral, and dorsal types) and medial medullary infarction (MMI) subtypes (ventromedial, centromedial, and dorsomedial types). We compared clinical manifestations, complications, END (defined as an increase in NIHSS score ≥4 points or death), and 90-day functional outcomes (modified Rankin Scale, mRS) among different subtypes. Multivariate logistic regression analysis was performed to identify independent predictors of favorable outcomes (mRS ≤ 2).

**Results:**

Among 352 patients, the most prevalent subtypes were dorsolateral (29.8%), superficial lateral (18.8%), and dorsal (16.5%). Significant heterogeneity was observed in clinical manifestations and END, with the dorsomedial subtype demonstrating the highest rates of limb motor dysfunction (65%), consciousness disturbance (27.5%), and END (25.0%). Regarding 90-day outcomes, the dorsomedial subtype had highest rate of poor favorable outcomes (65%), while the oblique lateral subtype had lowest rate of poor favorable outcomes (16.7%). The admission NIHSS score, age, dysphagia, contralateral limb involvement, and large-artery atherosclerosis were independent predictors of 90-day functional outcomes.

**Conclusion:**

This study demonstrated significant heterogeneity in clinical characteristics and 90-day outcomes among the seven anatomical subtypes of medullary infarction. The certain subtypes (particularly the dorsomedial subtype) were associated with higher rates of early neurological deterioration and poor functional outcomes.

## Introduction

The medulla oblongata resembles an inverted cone, which constitutes merely 0.1% of total brain volume and contains numerous critical neural pathways and nuclei, including the corticospinal tract, corticonuclear tract, medial lemniscus, hypoglossal nucleus, nucleus ambiguus, accessory nucleus, and spinal trigeminal nucleus (1). The medulla oblongata functions as a vital center in the human body, containing key respiratory control nuclei such as the dorsally located dorsal respiratory group (DRG) and the ventromedially situated ventral respiratory group (VRG). It also includes cardiovascular regulatory centers governed by the nucleus tractus solitarius and nucleus ambiguus, which collectively regulate respiratory rhythm and cardiac function. The medulla oblongata is highly vascularized, with significant anatomical variations, primarily supplied by the vertebral artery (2). Due to the medulla oblongata’s blood supply from numerous branch vessels and the high prevalence of vascular variations, the incidence of medullary infarction is lower than that of infarctions in other brainstem regions, representing only 7% of cases (3).

Based on the anatomical distribution of infarcted regions, medullary infarctions have traditionally been classified into medial medullary infarction (MMI) and lateral medullary infarction (LMI), with other rare types including bilateral medial medullary infarction (4) and hemimedullary infarction, which combines the clinical features of both lateral and medial medullary infarctions due to simultaneous involvement of both territories (5, 6). Due to the medulla oblongata’s complex anatomical structure and blood supply, infarctions in different regions manifest with diverse and complex clinical presentations, exhibiting significant variations in neurological signs (Supplementary Table S1). Lateral medullary infarction (Wallenberg syndrome) typically presents with ipsilateral facial sensory loss, contralateral body pain and temperature sensation loss, vertigo, ataxia, dysphagia, hoarseness, and Horner syndrome (7–9). Medial medullary infarction (Dejerine syndrome) is characterized by contralateral hemiparesis, contralateral deep sensation loss, and ipsilateral tongue paralysis (10, 11). These may present as various syndromes, including Jackson’s syndrome, Dejerine’s syndrome (medial medullary syndrome), Wallenberg’s syndrome (lateral medullary syndrome), hemimedullary syndrome, and Avellis syndrome (nucleus ambiguus-spinothalamic tract paralysis syndrome) (12). Clinically, these are often misdiagnosed as Guillain-Barré syndrome, brainstem encephalitis, or high cervical cord lesions, particularly in patients with initially negative diffusion-weighted imaging (DWI) findings during early disease onset. Furthermore, the prognosis of medullary infarction varies considerably, with some patients developing respiratory failure or sudden death due to involvement of respiratory and cardiovascular centers.

Previous studies on medullary infarction across different anatomical subtypes have been limited, primarily employing binary classifications, non-granular categorization, and single-center designs. Several anatomical classification systems for medullary infarction have been proposed. Vuilleumier et al. (13) described a six-subtype classification based on clinical and MRI correlations, categorizing lesions into lateral, dorsolateral, dorsal, ventromedial, paramedian, and dorsal territories. Kim (14) described patterns of lateral medullary infarction in 130 patients, while Kim and Han (15) categorized medial medullary infarction into three subtypes (ventromedial, middle, and dorsomedial). However, these classifications have not been widely adopted in clinical practice, and the relationship between specific anatomical subtypes and clinical outcomes remains incompletely understood. Previous studies were limited by small sample sizes, single-center designs, and lack of standardized outcome assessment.

Therefore, this study used a multicenter retrospective cohort approach to collect cases of acute isolated medullary infarction across various anatomical subtypes. We systematically analyzed risk factors, clinical characteristics, and clinical outcome for medullary infarctions in different locations, aiming to provide evidence-based support for early identification, clinical manifestations, and prognostic assessment of medullary infarction under distinct anatomical classifications.

## Materials and methods

### Study subjects

This multicenter retrospective cohort study enrolled patients with acute medullary infarction confirmed by DWI, demonstrating diffusion restriction with corresponding hypointensity on apparent diffusion coefficient (ADC) maps. Participants were hospitalized at three centers: First Affiliated Hospital of Kunming Medical University, West China Hospital of Sichuan University, and Taiyuan Hospital of Peking University First Hospital, between January 1, 2019, and October 31, 2024. The study protocol was approved by the Ethics Committee of the First Affiliated Hospital of Kunming Medical University [Approval No. (2024) Ethical L-239].

Inclusion Criteria: (1) Aged ≥18 years. (2) Admitted to the hospital within ≤7 days of onset. (3) Confirmed by cranial DWI showing diffusion restriction with corresponding low signal on ADC maps.

Exclusion Criteria: (1) Patients lost to follow-up 3 months after onset. (2) Lesions of other natures (e.g., pure hemorrhagic, inflammatory, demyelinating, and neoplastic lesions, etc.). (3) Concurrent infarctions in other locations, such as lesions in the parietal lobe, frontal lobe, cerebellum, and other regions. (4) Patients with severe missing clinical data. (5) Patients with residual severe neurological deficits (such as severe limb dysfunction and sensory disturbance) from previous strokes.

### Collection of baseline data for patients

We collected the following baseline data for the enrolled patients: (1) Demographic characteristics, including patients’ name, age and ethnicity. (2) Cerebrovascular disease risk factors and related medical history, such as history of previous cerebral infarction, previous intracerebral hemorrhage, hypertension, hyperlipidemia, transient ischemic attack (TIA), diabetes mellitus, atrial fibrillation, coronary heart disease, smoking, and alcohol consumption; family history, personal history; medication status (medication history and current medication use); and thrombolysis or interventional surgery. (3) Physical examination findings, including baseline vital signs, systolic and diastolic blood pressure at admission, and neurological specialist examination. (4) Baseline laboratory data, including complete blood count, urinalysis, liver function tests, renal function tests, lipid profile, fasting blood glucose, uric acid, and coagulation studies. (5) Cardiac and hemodynamic examinations, including 12-lead electrocardiogram, 24-h ambulatory electrocardiogram monitoring, and 24-h ambulatory blood pressure monitoring should be completed during hospitalization.

### Assessment of clinical data

The etiological classification and clinical scale scoring of all patients included in the study were evaluated by three board-certified neurologists (with 5–15 years of experience in stroke management). The etiological classification was determined based on the criteria of the Trial of Org 10,172 in Acute Stroke Treatment (TOAST) (16, 17), with partial modifications to the diagnostic criteria for the large-artery atherosclerosis subtype: (1) presence of at least one traditional risk factor for atherosclerosis; (2) intracranial large artery stenosis of mild degree or higher (corresponding to the vertebral artery in this study). Patients meeting the above criteria were classified as the large-artery atherosclerosis subtype, while the criteria for other subtypes remained consistent with the traditional TOAST classification. The severity of the disease was assessed using the National Institutes of Health Stroke Scale (NIHSS). Dysphagia was assessed using the Water Swallow Test.

### Imaging examinations

All patients underwent standardized neurovascular imaging during hospitalization. Endocranial magnetic resonance imaging (MRI) was performed using Philips Achieva 1.5T or 3.0T scanners (Philips Medical Systems, Best, Netherlands), including the following sequences: diffusion-weighted imaging (DWI) with *b*-values of 0 and 1,000 s/mm^2^, susceptibility-weighted imaging (SWI), T1-weighted (T1W), T2-weighted (T2W), T2-weighted fluid-attenuated inversion recovery with fat saturation (T2W-FLAIR-FS), and three-dimensional time-of-flight magnetic resonance angiography (3D-TOF MRA). Diffusion restriction was defined as hyperintensity on DWI (*b* = 1,000 s/mm^2^) with corresponding hypointensity on apparent diffusion coefficient (ADC) maps to exclude T2 shine-through artifacts. Computed tomography angiography (CTA) was performed using GE Revolution CT following intravenous injection of iodinated contrast (80–100 mL at 4–5 mL/s). Carotid vascular ultrasound was performed using color Doppler to assess the carotid and vertebral arteries. Chest computed tomography was also performed when clinically indicated. All imaging data were independently reviewed by two neuroradiologists with more than 5 years of experience in neurovascular imaging, with discrepancies resolved through consensus. To ensure consistency across the three participating centers, all MRI scanners underwent regular standardized calibration, and each center adhered to unified scanning protocols and image reconstruction parameters.

### Classification criteria

**Figure 1:**
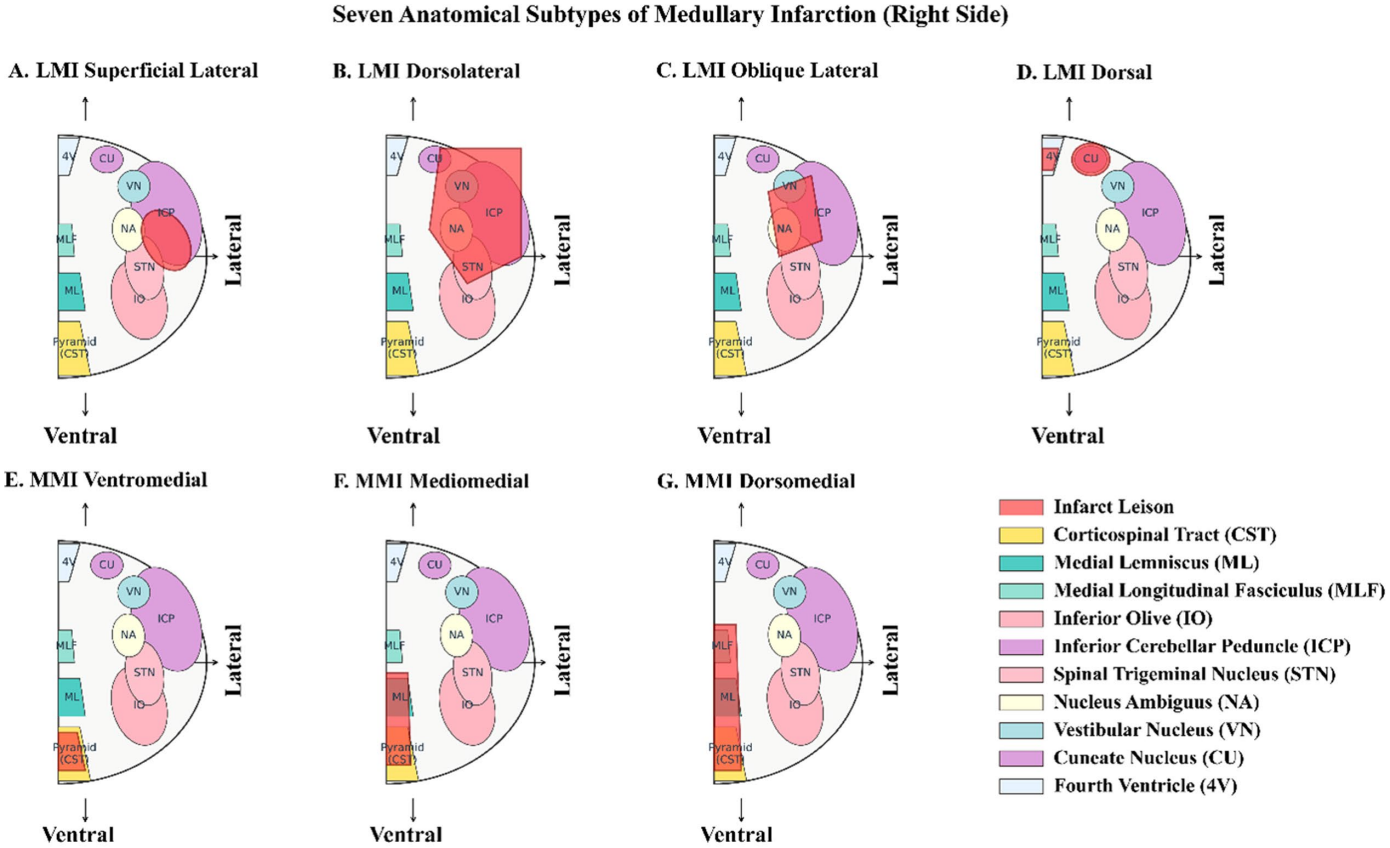
Seven anatomical subtypes of medullary infarction: schematic representation of lesion topography and neuroanatomical correlates (right side). Diagrammatic axial sections of the medulla oblongata illustrate the spatial distribution of infarct lesions (red shaded areas) relative to key neuroanatomical structures. **(A)** Lateral medullary infarction (LMI) superficial lateral: a small superficial lesion in the lateral medulla involving the inferior cerebellar peduncle (ICP) and spinal trigeminal nucleus (STN). **(B)** LMI dorsolateral: an extensive lesion involving the dorsolateral region, affecting the vestibular nucleus (VN), ICP, STN, and nucleus ambiguus (NA). **(C)** LMI oblique lateral: a characteristic oblique lesion extending from dorsolateral to ventrolateral, involving NA, STN, and ICP. **(D)** LMI dorsal: a small dorsal lesion near the floor of the fourth ventricle (4V), involving the cuneate nucleus (CU) and VN. **(E)** Medial medullary infarction (MMI) ventromedial: a ventromedial lesion involving the corticospinal tract (CST) in the pyramid. **(F)** MMI mediomedial: an intermediate medial lesion involving CST and medial lemniscus (ML). **(G)** MMI dorsomedial: An extensive dorsomedial lesion involving CST, ML, and medial longitudinal fasciculus (MLF). LMI, lateral medullary infarction; MMI, medial medullary infarction; 4V, fourth ventricle; CU, cuneate nucleus; VN, vestibular nucleus; NA, nucleus ambiguus; ICP, inferior cerebellar peduncle; STN, spinal trigeminal nucleus; ML, medial lemniscus; MLF, medial longitudinal fasciculus; CST, corticospinal tract.

This classification system was based on the anatomical location of infarct lesions observed on MRI-DWI images, primarily derived from the studies by Vuilleumier et al. (13), Kim (14) and Kim et al. (18), with subsequent refinement and expansion (Figure 1, Supplementary Table S1).

(1) Superficial lateral type: the infarct lesion is typically located in the caudal medulla, confined to the superficial lateral region, primarily involving the lateral portion of the inferior cerebellar peduncle and superficial fibers of the spinothalamic tract (Figure 1A) (13, 14).
(2) Dorsolateral type: the infarct lesion involves the entire dorsolateral region of the medulla, extending from lateral to dorsal, representing the most extensive subtype of lateral medullary infarction (Figure 1B) (13, 14).
(3) Oblique lateral type: the infarct lesion exhibits a characteristic oblique band-like pattern on MRI, extending from the dorsolateral to anterolateral medulla, primarily involving the nucleus ambiguus, spinal trigeminal nucleus, and spinothalamic tract (Figure 1C) (14, 19).
(4) Dorsal type: the infarct lesion is confined to the dorsal medulla, near the floor of the fourth ventricle, primarily involving the gracile nucleus, cuneate nucleus, and associated visceral nerve nuclei (Figure 1D) (18).
(5) Ventromedial type: the infarct lesion is restricted to the ventral portion of the medial medulla, primarily involving the corticospinal tract (pyramidal tract) and hypoglossal nucleus (Figure 1E) (15, 20).
(6) Mediomedial type: the infarct lesion extends from the ventral to intermediate regions of the medulla, primarily involving the corticospinal tract and medial lemniscus, sparing the medial longitudinal fasciculus (Figure 1F) (15, 21).
(7) Dorsomedial type: the infarct lesion extends further dorsally, encompassing extensive regions of the medial medulla, including the corticospinal tract, medial lemniscus, and medial longitudinal fasciculus, representing the most extensive subtype of medial medullary infarction (Figure 1G) (15, 20, 22).

### Clinical outcome

Short-term indicators: (1) NIHSS score and mRS score at discharge. (2) Early neurological deterioration (END), defined as an increase of ≥2 points in NIHSS score within 5 days after admission (23, 24). Long-Term indicators: 90-day mRS score. All participants underwent standardized telephone follow-up at 90 days post-onset. Each patient’s mRS score was evaluated by two experienced neurologists without knowledge of the patients’ baseline data. An mRS score of 0–2 at 90 days was classified as a favorable functional outcome, while a score of 3–6 at 90 days was classified as an unfavorable functional outcome.

### Statistical analysis

All statistical analyses were performed using SPSS Statistics version 26.0. Normality tests were conducted for continuous variables. Normally distributed continuous data were expressed as mean ± standard deviation (mean ± SD), whereas non-normally distributed data were presented as median (first quartile, third quartile) [M (Q1, Q3)]. Between-group comparisons were performed using independent samples t-test, Mann–Whitney *U* test, as appropriate. Categorical variables were expressed as frequency (percentage) [*n* (%)], and group comparisons were conducted using chi-square test or continuity-corrected chi-square test. To compare the seven anatomical subtypes, one-way analysis of variance (ANOVA) followed by Tukey’s *post hoc* test was used for normally distributed continuous variables, while the Kruskal–Wallis *H* test followed by Dunn’s *post hoc* test with Bonferroni correction was employed for non-normally distributed variables. Comparisons of categorical variables were performed using chi-square test or Fisher’s exact test. Multivariate logistic regression analysis was conducted to identify independent predictors of 90-day poor outcomes. Age and baseline NIHSS score were forced into the model. Other significant variables (those with *p* < 0.05 in univariate analysis and considered clinically relevant) were also entered into the multivariate logistic regression model. A two-tailed *p*-value < 0.05 was considered statistically significant.

## Results

### Description of all enrolled patients

**Figure 2:**
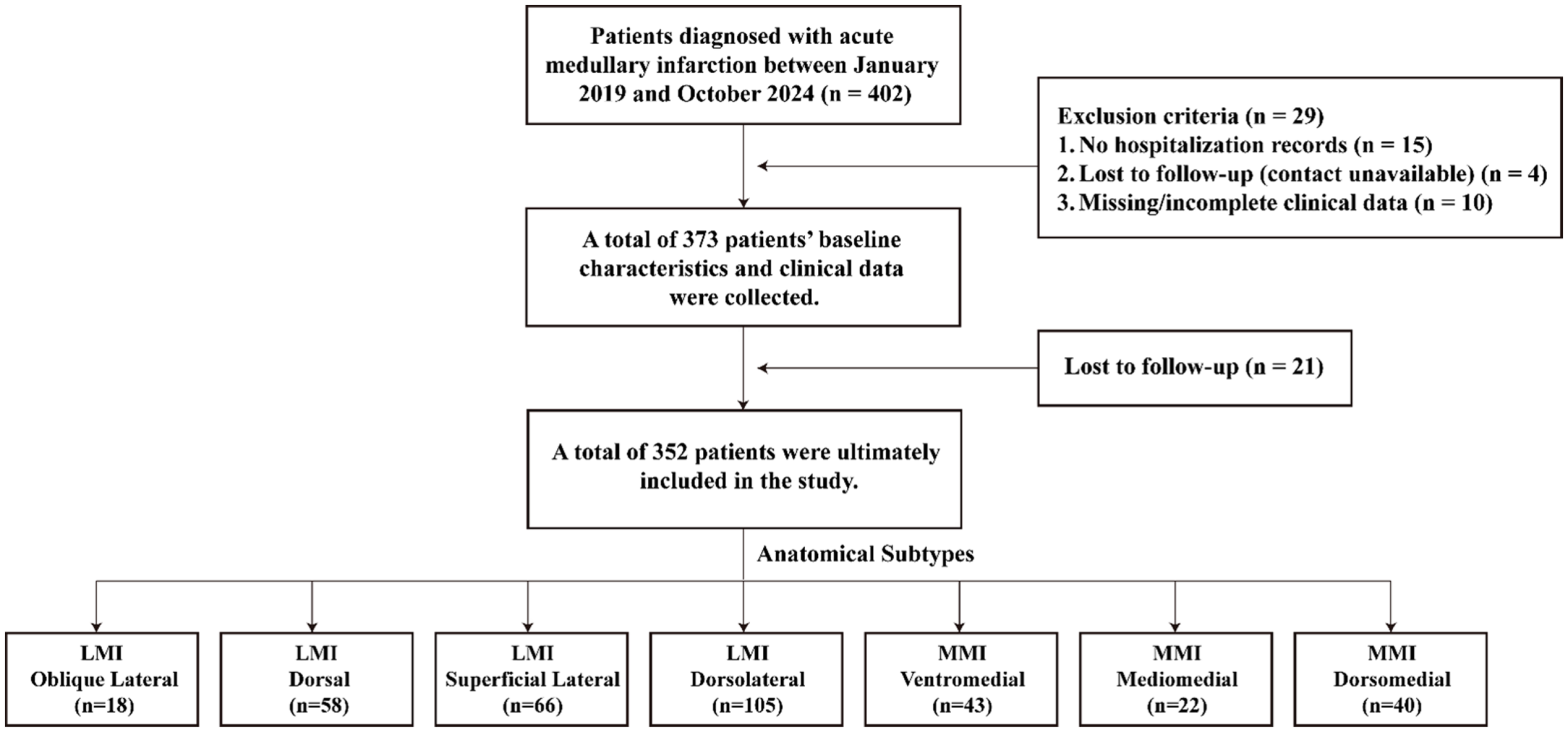
Enrollment of cohort study participants.

A total of 402 patients with acute medullary infarction were initially identified during the study period. After excluding 15 cases without hospitalization records, 4 cases with lost contact information, and 10 cases with missing clinical data, 373 patients were included in the baseline analysis. During the 90-day follow-up for clinical outcomes, 21 patients were lost to follow-up. Ultimately, 352 eligible patients were included in the final analysis, comprising 170 cases from First Affiliated Hospital of Kunming Medical University, 117 from West China Hospital of Sichuan University, and 65 from Taiyuan Hospital of Peking University First Hospital (Figure 2). Of the 352 patients included in the cohort study, 66 were female (18.80%) and 286 were male (81.30%), with a mean age of 56.5 ± 14.6 years.

### Overall characteristics

Regarding the transverse location of medullary infarction, the incidence rates in descending order were as follows: dorsolateral (29.80%), superficial lateral (18.80%), dorsal (16.50%), ventromedial (12.20%), dorsomedial (11.40%), mediomedial (6.30%), and oblique lateral (5.10%). Based on the TOAST classification, the most common etiology of medullary infarction was large-artery atherosclerosis, accounting for 199 cases (56.53%), followed by small-vessel occlusion in 108 cases (30.68%), and other determined etiologies in 45 cases (12.78%).

The prevalent clinical manifestations and symptoms observed in patients with medullary infarction, in descending order of frequency, were: sensory disturbance (75.00%), dizziness (62.22%), limb motor impairment (56.82%), ataxia (51.70%), vomiting (42.05%), dysarthria (43.75%), dysphagia (36.08%), facial paralysis (34.94%), vertigo (34.38%), headache (28.77%), nystagmus (19.89%), hiccups (10.80%), diplopia (9.38%), altered consciousness (8.52%), and ocular movement dysfunction (7.10%).

### Comparison of baseline characteristics based on the transverse classification

Demographic of medullary infarction were compared based on the transverse classification (Table 1). The ventromedial group exhibited the highest age of onset, whereas the dorsolateral group demonstrated the lowest age of onset (*p* = 0.002). The mediomedial group showed a higher prevalence of hypertension (*p* = 0.004) and diabetes mellitus (*p* = 0.017) among patients.

**Table 1:** Baseline characteristics by transverse classification of acute medullary infarction.

Variable	Distribution of transverse classifications in acute medullary infarction, *n* (%)							*p*-Value
	LMI oblique lateral (*n* = 18)	LMI dorsal (*n* = 58)	LMI superficial lateral (*n* = 66)	LMI dorsolateral (*n* = 105)	MMI ventromedial (*n* = 43)	MMI mediomedial (*n* = 22)	MMI dorsomedial (*n* = 40)	
Demographic characteristics								
Age	54.8 ± 15.4	55.8 ± 15.7	59.2 ± 14.3	51.9 ± 14.4	60.7 ± 12.7	59.4 ± 14.6	60.5 ± 13.1	0.002^*^
Gender								0.093
Male	16 (88.9)	41 (70.7)	50 (75.8)	86 (81.9)	37 (86.0)	21 (95.5)	35 (87.5)	
Female	2 (11.1)	17 (29.3)	16 (24.2)	19 (18.1)	6 (14)	1 (4.5)	5 (12.5)	
Cerebrovascular risk factors								
Hypertension	11 (61.1)	37 (63.8)	51 (77.3)	56 (53.3)	31 (72.1)	19 (86.4)	31 (77.5)	0.004^*^
Diabetes mellitus	7 (38.9)	19 (32.8)	24 (36.4)	23 (21.9)	21 (48.8)	12 (54.5)	15 (37.5)	0.017^*^
Clinical manifestations								
Sensory disturbance								<0.001^*^
No	5 (27.8)	15 (25.9)	26 (39.4)	18 (17.1)	15 (34.9)	4 (18.2)	5 (12.8)	
Crossed type	3 (16.7)	12 (20.7)	10 (15.2)	30 (28.6)	2 (4.7)	2 (9.1)	2 (5.1)	
Contralateral face and limb	3 (16.7)	3 (5.2)	3 (4.5)	7 (6.7)	5 (11.6)	2(9.1)	5 (12.8)	
Ipsilateral face	1 (5.6)	8 (13.8)	3 (4.5)	9 (8.6)	0 (0.0)	1 (4.5)	0 (0.0)	
Ipsilateral limb	0 (0.0)	4 (6.9)	7 (10.6)	11 (10.5)	1 (2.3)	0 (0.0)	1 (2.6)	
Contralateral limb	6 (33.3)	6 (10.3)	14 (21.2)	18 (17.1)	17 (39.5)	11 (50)	22 (56.4)	
Ipsilateral face and limb	0 (0.0)	9 (15.5)	3 (4.5)	11 (10.5)	2 (4.7)	1(4.5)	5 (12.8)	
Bilateral limbs	0 (0.0)	1 (1.7)	0 (0.0)	1 (1.0)	1 (2.3)	1 (4.5)	0 (0.0)	
Limb motor dysfunction								<0.001^*^
No	13 (72.2)	30 (51.7)	36 (54.5)	52 (49.5)	10 (23.3)	6 (27.3)	5 (12.5)	
Ipsilateral limb	1 (5.6)	19 (32.8)	19 (28.8)	32 (30.5)	2 (4.7)	0 (0.0)	1 (2.5)	
Contralateral limb	4 (22.2)	2 (3.4)	9 (13.6)	12 (11.4)	30 (69.8)	14 (63.6)	26 (65)	
Tetraplegia	0 (0.0)	2 (3.4)	2 (3.0)	7 (6.7)	1 (2.3)	1 (4.5)	8 (20.0)	
Others	0 (0.0)	5 (8.6)	0 (0.0)	2 (1.9)	0 (0.0)	1 (4.5)	0 (0.0)	
Dysarthria	9 (50.0)	19 (32.8)	18 (27.3)	54 (51.4)	14 (32.6)	10 (45.5)	30 (75.0)	<0.001^*^
Dysphagia	12 (66.7)	20 (34.5)	16 (24.2)	48(45.7)	8 (18.6)	4 (18.2)	19 (47.5)	<0.001^*^
Facial Paralysis	7 (38.9)	24 (41.4)	14 (21.2)	43 (41.0)	10 (23.3)	7 (31.8)	18 (45.0)	0.044^*^
Ataxia	7 (38.9)	40 (69.0)	38 (57.6)	62 (59)	13 (30.2)	6 (27.3)	16 (40.0)	<0.001^*^
Dizziness	13 (72.2)	5 (60.3)	42 (63.6)	71 (67.6)	27 (62.8)	12 (54.5)	19 (47.5)	0.383
Vertigo	7 (38.9)	22 (37.9)	26 (39.4)	40 (38.1)	8 (18.6)	5 (22.7)	13 (32.5)	0.244
Headache	4 (22.2)	21 (36.2)	24 (36.4)	37 (35.2)	7 (16.3)	4 (18.2)	4 (10.0)	0.008^*^
Diplopia	1 (5.6)	7 (12.1)	3 (4.5)	14 (13.3)	2 (4.7)	2 (9.1)	4 (10.0)	0.484
Nystagmus	4 (22.2)	12 (20.7)	9 (13.6)	31 (29.5)	3 (7.0)	2 (9.1)	9 (22.5)	0.028^*^
Vomiting	7 (38.9)	29 (50.0)	29 (43.9)	48 (45.7)	12 (27.9)	7 (31.8)	16 (40.0)	0.340
Hiccup	1 (5.6)	9 (15.5)	6 (9.1)	19 (18.1)	1 (2.3)	0 (0.0)	2 (5)	0.021^*^
Consciousness status								0.014^*^
Alert	17 (94.4)	56 (96.6)	63 (95.5)	96 (91.4)	40 (93)	21 (95.5)	29 (72.5)	
Somnolence	1 (5.6)	1 (1.7)	0 (0.0)	7(6.7)	1 (2.3)	1 (4.5)	8 (20.0)	
Coma	0 (0.0)	1 (1.7)	2 (3.0)	1 (1)	1 (2.3)	0 (0.0)	1 (2.5)	
Confusion	0 (0.0)	0 (0.0)	0 (0.0)	1 (1.0)	1 (2.3)	0 (0.0)	2 (5.0)	
Delirium	0 (0.0)	0 (0.0)	1 (1.5)	0 (0.0)	0 (0.0)	0 (0.0)	0 (0.0)	
Ocular movement disorder	1 (5.6)	2 (3.4)	2 (3.1)	10 (9.5)	3 (7)	0 (0.0)	7 (17.5)	0.093
Complications								
Respiratory tract infection	3 (17.6)	12 (20.7)	11 (16.9)	29 (27.6)	4 (9.3)	3 (13.6)	18 (45)	0.003^*^
Urinary retention	0 (0.0)	0 (0.0)	6 (9.2)	8 (7.6)	2 (4.7)	1 (4.5)	7 (17.5)	0.028^*^
Electrolyte disturbance	2 (11.8)	18 (31.0)	17 (26.2)	16 (15.4)	8 (18.6)	6 (27.3)	16 (41)	0.025^*^
Cholesterol, mmol/L, $\bar{x}$ ± s	4.5 ± 1.2	4.1 ± 1.1	4.3 ± 1.2	4.2 ± 1.2	3.7 ± 1.5	5 ± 1.8	4.3 ± 1.3	0.010^*^
TOAST								0.242
Small artery disease	6 (33.33)	20 (34.48)	21 (31.82)	24 (22.86)	21 (48.84)	6 (27.27)	10 (25.00)	
Large artery atherosclerosis	10 (55.56)	29 (50.00)	37 (56.06)	64 (60.95)	18 (41.86)	13 (59.09)	28 (70.00)	
Other	2 (11.11)	9 (15.52)	8 (12.12)	17 (16.19)	4 (9.30)	3 (13.64)	2 (5.00)	
NIHSS								
Admission	2.33 ± 1.37	2.83 ± 2.06	2.45 ± 1.55	3.55 ± 1.91	3.74 ± 3.37	3.73 ± 2.05	8.55 ± 8.06	<0.001^*^
Discharge	3.50 ± 6.71	2.38 ± 1.68	2.59 ± 4.31	3.42 ± 3.67	4.17 ± 6.12	3.00 ± 1.35	9.25 ± 9.97	<0.001^*^

^*^Indicates *p* < 0.05, LMI, lateral medullary infarction; MMI, medial medullary infarction; NIHSS, National Institutes of Health Stroke Scale.

### Comparison of clinical characteristics based on the transverse classification

Significant differences were observed in the incidence of symptoms, signs, and complications among medullary infarctions of different transverse locations (Table 1). Regarding symptoms and signs, the sensory disturbance was highest incidence of in the dorsomedial type and lowest incidence in superficial lateral type (*p* < 0.001). The limb motor disturbance had the highest incidence in the dorsomedial type and the lowest in the oblique lateral type (*p* < 0.001). Dysphagia had the highest incidence in the oblique lateral type and the lowest in the mediomedial type (*p* < 0.001), while ataxia was most prevalent in the dorsal type and least prevalent in the mediomedial type (*p* < 0.001). Headache showed the highest incidence in the superficial lateral type and the lowest in the dorsomedial type (*p* = 0.008), and hiccups were most common in the dorsolateral type and least common in the mediomedial type (*p* = 0.006). Nystagmus had the highest incidence in the dorsolateral type and the lowest in the ventromedial type (*p* = 0.028), and facial paralysis was most frequent in the dorsomedial type and least frequent in the superficial lateral type (*p* = 0.047). Additionally, among patients with disturbance of consciousness, the dorsomedial type had the highest proportion of this condition (*p* = 0.014).

### Comparison complications based on the transverse classification

For complications (Table 1), the incidence of respiratory tract infection was the highest in the dorsomedial type (45.00% in dorsomedial vs. 27.60% in dorsolateral) and the lowest in the ventromedial type (*p* = 0.003); urinary retention had the highest incidence in the dorsomedial type and the lowest in the oblique lateral and dorsal types (*p* = 0.028); and electrolyte disturbance was most common in the dorsomedial type and least common in the oblique lateral type (*p* = 0.025). Cholesterol levels also showed significant differences in patients with medullary infarction across different transverse locations (*p* = 0.010), with the mediomedial type exhibiting the highest cholesterol levels.

### Comparison of imaging sagittal layers based on the transverse classification

Significant differences were observed in the distribution of different sagittal groups among the transverse groups (*p* < 0.001) (Table 2). For the LMI oblique lateral group, the highest proportion was in the middle sagittal group (55.56%). For the LMI dorsal group, the highest proportion was in the middle sagittal group (41.38%). For the LMI superficial lateral group, the highest proportion was in the middle sagittal group (43.94%). For the LMI dorsolateral group, the highest proportion was in the middle sagittal group (42.86%). For the MMI ventromedial group, the highest proportion was in the upper sagittal group (51.16%), while the lowest proportion was in the lower sagittal group (2.33%); similarly, the MMI dorsomedial group also had a low proportion in the lower sagittal group (2.50%). For the MMI mediomedial group, the highest proportion was in the upper sagittal group (68.18%), while the lowest proportion was in the lower sagittal group (0.00%). For the MMI dorsomedial group, the highest proportion was in the upper sagittal group (50.00%), while the lowest proportion was in the lower sagittal group (2.50%). These grouping results indicated that medial medullary infarctions primarily involved the upper part of the medulla oblongata, whereas lateral medullary infarctions predominantly involved the middle part.

**Table 2:** Comparison results of sagittal classifications in acute medullary infarction.

Sagittal classification	Transverse classification, *n* (%)							Total	*p*-Value
	LMI oblique lateral	LMI dorsal	LMI superficial lateral	LMI dorsolateral	MMI ventromedial	MMI mediomedial	MMI dorsomedial		
Upper part	5 (27.78)	16 (27.59)	9 (13.64)	9 (8.57)	22 (51.16)	15 (68.18)	20 (50.00)	96 (27.27)	*p* < 0.001^*^
Middle part	10 (55.56)	24 (41.38)	29 (43.94)	45 (42.86)	7 (16.28)	3 (13.64)	10 (25.00)	128 (36.36)	
Lower part	0 (0.00)	2 (3.45)	14 (21.21)	4 (3.81)	1 (2.33)	0 (0.00)	1 (2.50)	22 (6.25)	
Upper-middle part	1 (5.56)	7 (12.07)	3 (4.55)	23 (21.90)	9 (20.93)	1 (4.55)	5 (12.50)	49 (13.92)	
Middle-lower part	1 (5.56)	7 (12.07)	10 (15.15)	18 (17.14)	2 (4.65)	1 (4.55)	3 (7.50)	42 (11.93)	
Upper-middle-lower part	1 (5.56)	2 (3.45)	1 (1.52)	6 (5.71)	2 (4.65)	2 (9.09)	1 (2.50)	15 (4.26)	
Total	18	58	66	105	43	22	40	352	

^*^Indicates *p* < 0.05, LMI, lateral medullary infarction; MMI, medial medullary infarction.

For LMI, except for the oblique lateral type, the highest proportion of cases with poor prognosis was in the middle sagittal group. Among these, for the dorsolateral type with poor prognosis, the proportion of cases involving multiple segments (in addition to the middle part) was also relatively high. In contrast, MMI cases with poor prognosis were mostly concentrated in the upper sagittal group (Table 3, Supplementary Figure S1).

**Table 3:** The poor prognosis by sagittal and transverse classifications of acute medullary infarction.

Sagittal classification	Transverse classification, *n* (%)							Total
	LMI oblique lateral	LMI dorsal	LMI superficial lateral	LMI dorsolateral	MMI ventromedial	MMI mediomedial	MMI dorsomedial	
Upper part	2 (5.4)	5 (13.5)	1 (2.7)	4 (10.8)	8 (21.6)	7 (18.9)	10 (27)	37 (100)
Middle part	1 (2.1)	6 (12.8)	11 (23.4)	18 (38.3)	3 (6.4)	0 (0)	8 (17)	47 (100)
Lower part	0 (0.00)	1 (14.3)	4 (57.1)	2 (28.6)	0 (0)	0 (0.00)	0 (0)	7 (100)
Upper-middle part	0 (0.00)	2 (8.7)	1 (4.3)	10 (43.5)	5 (21.7)	1 (4.3)	4 (17.4)	23 (100)
Middle-lower part	0 (0.00)	3 (13.6)	5 (22.7)	10 (45.5)	1 (4.5)	0 (0)	3 (13.6)	22 (100)
Upper-middle-lower part	0 (0.00)	0 (0.00)	1 (12.5)	4 (50)	1 (12.5)	1 (12.5)	1 (12.5)	8 (100)
Total	3	17	23	48	18	9	26	144

### Comparison severity based on the transverse classification

Significant differences were observed in the distribution of admission mRS scores among the transverse groups (*p* < 0.001). The distribution of admission mRS scores was markedly uneven across the groups, with the dorsolateral and dorsomedial groups exhibiting a higher proportion of patients with severe and profound disability. Admission NIHSS scores also differed significantly among the transverse groups (*p* < 0.001) (Supplementary Table S2). The dorsomedial group demonstrated the highest admission NIHSS score (8.55 ± 8.06), whereas the oblique lateral group had the lowest score (2.33 ± 1.37).

### Comparison clinical outcome based on the transverse classification

Discharge mRS scores exhibited significant variation across the transverse groups (*p* < 0.001). Among patients with medullary infarction, the dorsomedial group displayed the poorest prognosis at discharge, followed by the dorsolateral group, while the oblique lateral group exhibited the most favorable prognosis. Discharge NIHSS scores also varied significantly among the groups (*p* < 0.001), with the dorsomedial group recording the highest score (9.25 ± 9.97) and the dorsal group the lowest (2.38 ± 1.68) (Table 1).

Significant differences were observed in the distribution of 90-day mRS scores among the transverse groups (*p* < 0.001). The dorsomedial group had the highest proportion of poor prognosis (65.00%), whereas the oblique lateral group had the lowest proportion (16.70%) (Table 4, Supplementary Figure S2).

**Table 4:** 90-day modified Rankin Scale (mRS) clinical prognosis scores.

3-Month mRS	LMI oblique lateral	LMI dorsal	LMI superficial lateral	LMI dorsolateral	MMI ventromedial	MMI mediomedial	MMI dorsomedial	Total	*p*-Value
0	2 (11.11)	6 (10.34)	10 (15.15)	4 (3.81)	7 (16.28)	0 (0.00)	0 (0.00)	29 (8.24)	*p* < 0.001^*^
1	7 (38.89)	15 (25.86)	17 (25.76)	19 (18.10)	9 (20.93)	3 (13.64)	4 (10.00)	74 (21.02)	
2	6 (33.33)	20 (34.48)	16 (24.24)	34 (32.38)	8 (18.60)	10 (45.45)	10 (25.00)	104 (29.55)	
3	1 (5.56)	13 (22.41)	12 (18.18)	31 (29.52)	11 (25.58)	7 (31.82)	7 (17.50)	82 (23.30)	
4	1 (5.56)	3 (5.17)	4 (6.06)	10 (9.52)	6 (13.95)	1 (4.55)	10 (25.00)	35 (9.94)	
5	0 (0.00)	0 (0.00)	0 (0.00)	0 (0.00)	1 (2.33)	0 (0.00)	6 (15.00)	7 (1.99)	
6	1 (5.56)	1 (1.72)	7 (10.61)	7 (6.67)	1 (2.33)	1 (4.55)	3 (7.50)	21 (5.97)	
Total	18	58	66	105	43	22	40	352	

^*^Indicates *p* < 0.05, mRS, modified Rankin Scale; LMI, lateral medullary infarction; MMI, medial medullary infarction.

**Figure 3:**
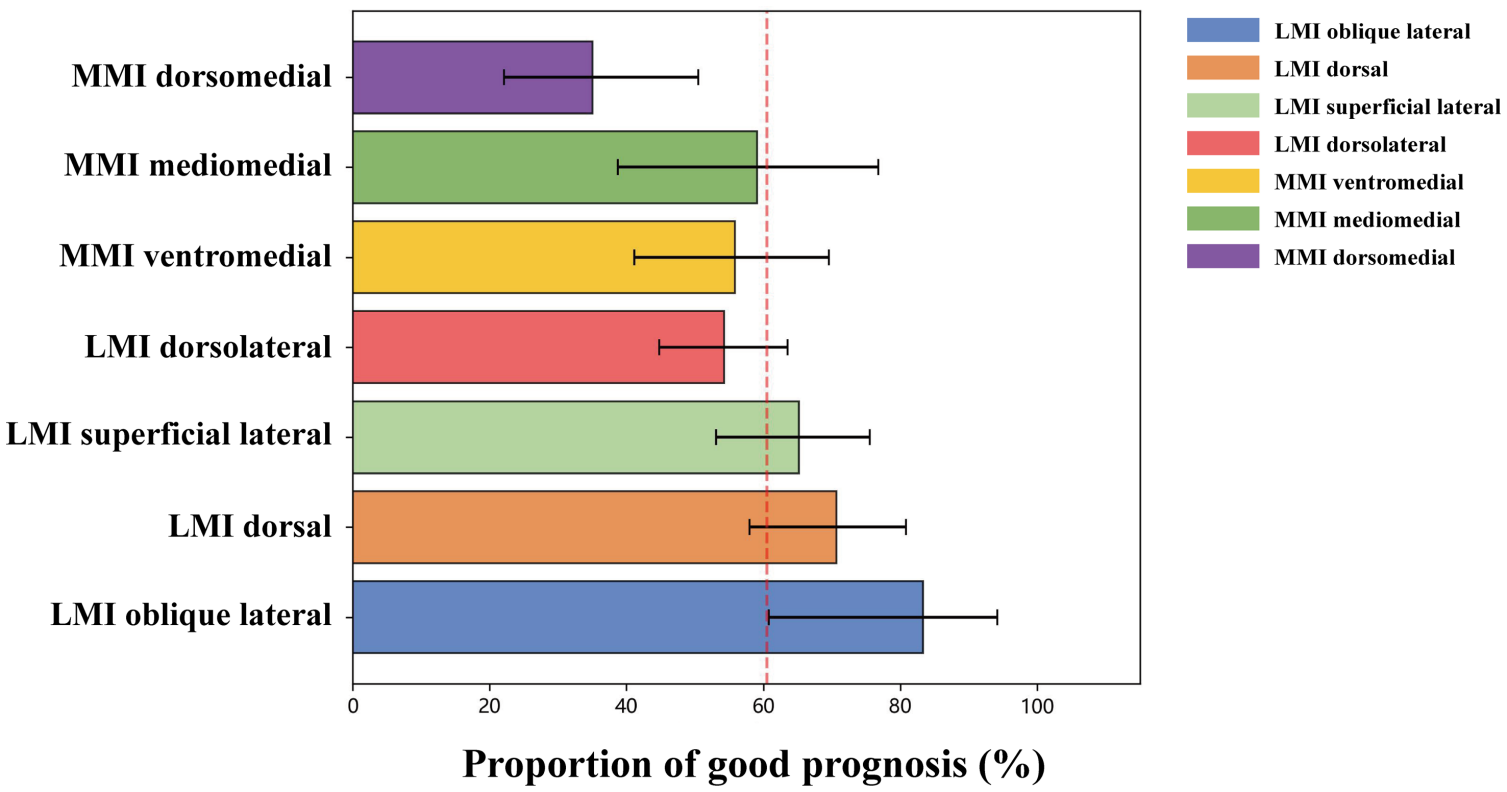
Proportion of good prognosis among different anatomical subtypes of medullary infarction. This figure visually presents the point estimates of 3-month favorable outcome rates (mRS ≤ 2) for each subtype and their uncertainty intervals, using the overall mean (approximately 60%, red dashed line) as the reference benchmark.

The LMI oblique lateral subtype exhibited the highest good outcome rate (approximately 83%), with the lower bound of its confidence interval substantially exceeding the overall mean, suggesting a statistically significant prognostic advantage. The LMI dorsal subtype followed (approximately 71.00%), also demonstrating significantly better outcomes. The LMI superficial lateral, LMI dorsolateral, MMI ventromedial, and MMI mediomedial subtypes showed prognoses comparable to the population average. In contrast, the MMI dorsomedial subtype demonstrated the lowest good outcome rate (approximately 35.00%), with its confidence interval entirely below the overall mean, indicating a significantly inferior prognosis (Figure 3).

### Early neurological deterioration

Among the 352 enrolled patients, 35 (9.90%) experienced END, yielding an overall incidence of 9.9%. Significant heterogeneity was observed across subtypes. The incidence of EDN differed significantly among the transverse groups (*p* = 0.015) (Table 5). The dorsomedial group had the highest incidence (25.00%), followed by the dorsolateral (12.38%) and ventromedial (11.63%) groups, while the mediomedial (4.55%) and superficial lateral (4.55%) groups exhibited the lowest incidence (Supplementary Figure S3, Table S3).

**Table 5:** Assessment of early neurological deterioration (END) in acute medullary infarction.

END	LMI oblique lateral (*n* = 18)	LMI dorsal (*n* = 58)	LMI superficial lateral (*n* = 66)	LMI dorsolateral (*n* = 105)	MMI ventromedial (*n* = 43)	MMI mediomedial (*n* = 22)	MMI dorsomedial (*n* = 40)	Total	*p*-Value
No	17 (94.44)	56 (96.55)	63 (95.45)	92 (87.62)	38 (88.37)	21 (95.45)	30 (75.00)	317 (90.06)	*p* < 0.05^*^
Yes	1 (5.56)	2 (3.45)	3 (4.55)	13 (12.38)	5 (11.63)	1 (4.55)	10 (25.00)	35 (9.94)	
Total	18	58	66	105	43	22	40	352	

^*^Indicates *p* < 0.05, END, early neurological deterioration; LMI, lateral medullary infarction; MMI, medial medullary infarction.

### Multivariate logistic regression analysis of 90-day mRS prognosis

In the multivariable logistic regression analysis, after forcing age and admission NIHSS into the model, variables with *p* < 0.01 in univariable analysis (Supplementary Table S4) were further selected for inclusion. A total of 21 variables were ultimately entered into the model, including treatment modality, early neurological deterioration, clinical symptoms, limb motor impairment subtypes, risk factors, laboratory indicators, axial classification, and TOAST classification.

Multivariate logistic regression analysis demonstrated that advanced age (OR = 0.969, 95% CI: 0.946–0.992, *p* = 0.010), elevated admission NIHSS score (OR = 0.600, 95% CI: 0.483–0.745, *p* < 0.001), presence of dysphagia (OR = 0.458, 95% CI: 0.240–0.875, *p* = 0.018), large-artery atherosclerosis (OR = 0.449, 95% CI: 0.225–0.898, *p* = 0.024), and intravenous thrombolysis or endovascular therapy (versus conservative treatment) (OR = 0.331, 95% CI: 0.123–0.893, *p* = 0.029) were significant predictors of decreased likelihood for favorable functional outcomes at 3 months. In contrast, contralateral limb involvement (OR = 2.894, 95% CI: 1.152–7.273, *p* = 0.024) emerged as an independent predictor associated with enhanced probability of favorable outcomes (Table 6).

**Table 6:** Multivariate analysis of factors influencing 90-day prognosis in medullary infarction (dependent variable: 90-day mRS).

Variable	*β* coefficient	SE	OR	OR (95%CI)	*p*-value
Age	−0.0314	0.0121	0.9691	0.969 (0.946–0.992)	0.010^*^
Admission NIHSS	−0.5109	0.1108	0.5999	0.600 (0.483–0.745)	<0.001^*^
Treatment modality	−1.105	0.5059	0.3312	0.331 (0.123–0.893)	0.029^*^
END	−0.8692	0.584	0.4193	0.419 (0.133–1.317)	0.137
Clinical symptoms					
Dysarthria	−0.3733	0.3371	0.6885	0.688 (0.356–1.333)	0.268
Dysphagia	−0.7802	0.3298	0.4583	0.458 (0.240–0.875)	0.018^*^
Facial palsy	0.1017	0.3288	1.1071	1.107 (0.581–2.109)	0.757
Vertigo	0.4202	0.3215	1.5222	1.522 (0.811–2.859)	0.191
Limb motor impairment subtypes (vs. none)					
Ipsilateral	0.4321	0.3937	1.5406	1.541 (0.712–3.333)	0.272
Contralateral	1.0626	0.4702	2.894	2.894 (1.152–7.273)	0.024^*^
Quadriplegia	−0.9087	0.8133	0.4031	0.403 (0.082–1.984)	0.264
Other	0.8765	1.0319	2.4026	2.403 (0.318–18.158)	0.396
Risk factors					
Coronary heart disease	−0.4012	0.527	0.6695	0.670 (0.238–1.881)	0.447
Laboratory indicators					
Hemoglobin	0.0082	0.0079	1.0083	1.008 (0.993–1.024)	0.295
Albumin	−0.0305	0.0423	0.9699	0.970 (0.893–1.054)	0.470
Fasting blood glucose	−0.0348	0.0489	0.9658	0.966 (0.878–1.063)	0.477
Low-density lipoprotein	0.0948	0.1423	1.0994	1.099 (0.832–1.453)	0.505
Transverse subtype (vs. MMI dorsomedial)					
LMI oblique lateral	1.3769	1.0647	3.9626	3.963 (0.492–31.932)	0.196
LMI dorsal	0.1096	0.7158	1.1158	1.116 (0.274–4.539)	0.878
LMI superolateral	−0.5308	0.6915	0.5882	0.588 (0.152–2.281)	0.443
LMI dorsolateral	−0.0683	0.6315	0.934	0.934 (0.271–3.220)	0.914
MMI ventromedial	−0.8738	0.7051	0.4174	0.417 (0.105–1.662)	0.215
MMI medial	−0.3563	0.7708	0.7002	0.700 (0.155–3.172)	0.644
TOAST classification (vs. small artery disease)					
Large-artery atherosclerosis	−0.8	0.3535	0.4493	0.449 (0.225–0.898)	0.024^*^
Other	−0.461	0.4912	0.6307	0.631 (0.241–1.652)	0.348

^*^Indicates *p* < 0.05, END, early neurological deterioration; LMI, lateral medullary infarction; MMI, medial medullary infarction; toast, Trial of Org 10,172 in acute stroke treatment classification.

## Discussion

Our study revealed that among the seven anatomical subtypes of medullary infarction, the dorsolateral type was the most prevalent (29.80%), followed by the superficial lateral (18.80%) and dorsal types (16.50%). In contrast, the oblique lateral type was the rarest (5.10%). Regarding functional outcomes, the dorsomedial subtype demonstrated the poorest prognosis with a 65.00% rate of poor outcomes at 90 days, whereas the oblique lateral subtype exhibited the most favorable prognosis with only 16.70% poor outcomes.

The distinct clinical manifestations observed across subtypes can be explained by the specific neuroanatomical structures involved in each subtype. The dorsomedial subtype, which demonstrated the worst prognosis, involves the most extensive anatomical region including the corticospinal tract, medial lemniscus, and medial longitudinal fasciculus. This extensive involvement explains the high incidence of limb motor dysfunction (65.00%), facial paralysis (45.00%), and consciousness disturbance (27.50%) observed in this subtype. Previous studies have similarly reported that extensive medial medullary lesions involving multiple structures are associated with severe clinical presentations and poor outcomes (10).

Conversely, the oblique lateral subtype, despite involving the nucleus ambiguus and spinal trigeminal nucleus, showed the best prognosis. This may be attributed to the relatively focal lesion distribution and the absence of critical motor pathway involvement. The characteristic oblique band-like lesion pattern primarily affects sensory and bulbar functions while sparing the major motor tracts, consistent with previous descriptions of this rare subtype (14, 19).

The dorsal subtype exhibited the highest incidence of ataxia (69.00%), consistent with its involvement of the inferior cerebellar peduncle and vestibular nuclei. This finding aligns with prior reports that dorsal medullary infarction produces a distinct syndrome of isolated central vestibulopathy characterized by prominent ataxia and vertigo (25). The superficial lateral subtype, limited to the superficial lateral region involving primarily the inferior cerebellar peduncle and spinocerebellar tract, demonstrated relatively mild clinical presentations with preserved motor function in most cases. This pattern is consistent with the original description by Kim (14), who reported that superficial lateral infarctions typically present with milder symptoms compared to more extensive dorsolateral lesions.

END emerged as a critical prognostic event in our study. The overall END incidence was 9.90%, but substantial heterogeneity existed across subtypes. The dorsomedial subtype showed the highest END rate (25.00%), approximately 2.5 times the overall incidence. This finding had important clinical implications, as patients with dorsomedial infarction require close monitoring during the acute phase. Previous studies had reported variable END rates in medullary infarction, with our findings providing more granular data across anatomical subtypes (24). The prognostic impact of END was dramatic—patients experiencing END had only a 20.00% rate of favorable outcomes compared with 63.10% in those without END—a difference exceeding 40 percentage points. Notably, among patients who experienced END, the dorsomedial, mediomedial, and oblique lateral subtypes all showed 0.00% favorable outcome rates, indicating that once early deterioration occurs in these subtypes, functional recovery becomes extremely limited. This finding is consistent with previous reports that early neurological deterioration serves as a critical predictor of poor long-term prognosis in stroke patients (26).

Admission NIHSS score demonstrated the strongest predictive effect, with each one-point increase associated with a 40.00% reduction in the probability of favorable outcome. This finding underscores the importance of baseline neurological severity in prognostication and is consistent with extensive stroke literature demonstrating the prognostic value of NIHSS across various stroke subtypes. Advanced age was also an independent predictor of poor outcome, with each additional year of age associated with a 3.00% reduction in favorable outcome probability. This is consistent with previous studies demonstrating reduced functional recovery capacity in elderly stroke patients and specifically in medullary infarction cohorts (27). Dysphagia emerged as a significant independent predictor, highlighting the prognostic importance of bulbar function impairment. This finding remained consistent across all sensitivity analyses, providing robust evidence for its clinical relevance. Previous studies have similarly identified dysphagia as an important prognostic factor in lateral medullary syndrome, with aspiration pneumonia being a major complication affecting outcomes (28, 29).

A notable finding of our study is that after adjusting for age, admission NIHSS, and other covariates, none of the six axial subtypes (when compared with dorsomedial as reference) demonstrated statistically significant differences in 90-day outcomes. This finding was consistent across all three sensitivity analyses (multicenter-adjusted, small-subtype-excluded, and rare-subtype-merged models). This result suggests that the prognostic differences observed among subtypes in univariate analysis are largely mediated through baseline neurological severity and other clinical characteristics rather than representing independent effects of anatomical location per se. In other words, while certain subtypes are associated with worse outcomes, this association is primarily explained by the more severe neurological deficits present at baseline in those subtypes. This finding has important implications for clinical practice: while anatomical classification provides valuable information about expected clinical manifestations and potential complications, baseline neurological severity (as captured by NIHSS) remains the dominant prognostic factor. Clinicians should therefore focus on comprehensive neurological assessment rather than relying solely on anatomical subtype for prognostication.

Large-artery atherosclerosis (LAA) was identified as an independent predictor of poor outcome compared with small-artery occlusion (SAO). This finding remained significant in two of three sensitivity analyses, with attenuation observed only in the multicenter-adjusted model, suggesting potential center-specific variations in TOAST classification distribution. The prognostic disadvantage of LAA can be attributed to several factors: LAA lesions typically result from atherosclerotic plaque formation in large arteries, leading to more extensive and hemodynamically significant stenosis. These patients are also at higher risk of recurrent stroke and other cardiovascular events. In contrast, SAO affects smaller penetrating arteries, resulting in more limited lesions and relatively preserved collateral circulation (17, 30).

In this study, the definition of the LAA subtype in the TOAST classification was partially modified. However, some classification systems, such as the Chinese Ischemic Stroke Subclassification (CISS), do not mandate stenosis exceeding 50% for an LAA diagnosis. This modification was necessary given the limitations of non-invasive imaging techniques such as MRA and CTA in detecting vertebral artery branch lesions, which complicates the differentiation between LAA and SAO compared to anterior circulation infarction (31, 32).

Our analysis of sagittal distribution revealed distinct patterns between lateral and medial medullary infarctions. Medial subtypes (ventromedial, mediomedial, dorsomedial) predominantly involved the upper medulla (51.20, 68.20, and 50.00%, respectively), whereas lateral subtypes primarily affected the middle medulla. This distribution pattern reflects the vascular anatomy of the medulla, where medial regions receive blood supply from perforating branches originating proximally, while lateral regions are supplied by more distal branches (33).

Poor prognosis cases in medial subtypes were concentrated in the upper sagittal group, consistent with previous reports that upper medullary infarction is associated with worse outcomes. A study by Makita et al. (30) found that upper medullary infarction was significantly associated with poor prognosis, consistent with the distribution of poor prognosis in MMI observed in this study. Upper segment infarctions are typically limited to the pontomedullary junction area, and MMI frequently involves the pyramidal tract, leading to severe limb dysfunction and consequently poorer prognosis. For LMI, middle segment involvement and multi-segment involvement were associated with a higher incidence of poor prognosis, primarily because middle segment infarctions may affect the inferior olivary nucleus and certain autonomic nuclei.

Our findings are generally consistent with previous reports while providing more granular insights through the seven-subtype classification. The study by Vuilleumier et al. (13) described a six-subtype classification based on clinical and MRI correlations, categorizing lesions into lateral, dorsolateral, dorsal, ventromedial, paramedian, and dorsal territories. Our classification builds upon this framework with refinements: the dorsolateral type corresponds to the lateral territory (including the superficial lateral type), the dorsal type to the floor of the fourth ventricle (involving the gracile nucleus, cuneate nucleus, and spinal trigeminal nucleus), the ventromedial type to the paramedian territory, and the dorsomedial type to the dorsal territory (including the mediomedial and ventromedial types). The lateral subtype classification was derived from the study by Kim (14): superficial lateral corresponds to lateral involvement, dorsolateral to typical plus ventral extension, oblique lateral to ventral involvement with characteristic oblique pattern, and dorsal to dorsal involvement. The medial subtype classification was based on the studies by Kim and Han (15): ventromedial involves ventral regions, mediomedial involves ventral plus middle regions, and dorsomedial involves ventral plus middle plus dorsal regions. The prognostic hierarchy we identified—dorsomedial (worst) to oblique lateral (best)—represents a novel contribution to the literature. Previous studies primarily compared medial versus lateral medullary infarction as broad categories, whereas our granular classification enables more precise risk stratification. The dorsomedial subtype exhibited the worst prognosis, consistent with the findings of Memmedova et al. (26), who reported high progression rates and poor outcomes in medial medullary infarction.

Our study has several strengths. The multicenter design with three large clinical centers enhances the generalizability of our findings. The relatively large sample size (*n* = 352) for a rare stroke subtype provides adequate statistical power for subtype comparisons. The comprehensive data collection, including detailed clinical manifestations, neuroimaging characteristics, and 90-day functional outcomes, enables robust prognostic analyses.

The sensitivity analyses we performed—multicenter-adjusted, small-subtype-excluded, and rare-subtype-merged models—demonstrate the robustness of our findings across different analytical approaches. The consistency of key prognostic factors (age, admission NIHSS, dysphagia) across all models provides confidence in their clinical relevance.

Several limitations should be acknowledged. First, the retrospective design introduces potential selection bias and limits our ability to establish causal relationships. Second, the modified TOAST classification criteria for LAA (using ≥mild stenosis rather than ≥50% stenosis) may affect comparability with studies using traditional criteria. Third, several subtypes had relatively small sample sizes (oblique lateral *n* = 18, mediomedial *n* = 22), which may limit statistical power and precision of effect estimates for these rare subtypes. Fourth, the seven-subtype classification system, particularly the novel terms “superficial lateral” “mediomedial,” and “oblique lateral” has not been standardized in the neuroanatomical literature. While this classification enables more precise anatomical localization, its comparability with previous studies may be limited. External validation in independent cohorts is necessary to confirm the reproducibility and clinical utility of this classification system. Fifth, the requirement for MRI confirmation with DWI evidence of diffusion restriction may have introduced selection bias toward patients with less severe presentations, as those with severe clinical deterioration or contraindications to MRI may have been excluded. This is reflected in the relatively high rate of favorable outcomes observed in our cohort (59.10% with mRS ≤ 2 at 90 days), which may not be generalizable to all patients with medullary infarction.

In conclusion, the dorsomedial subtype exhibited the worst prognosis with the highest rates of early neurological deterioration and poor functional outcomes, while the oblique lateral subtype demonstrated the most favorable prognosis. While anatomical classification provides valuable information about expected clinical manifestations, baseline neurological severity remains the dominant prognostic factor.

## Data Availability

The original contributions presented in the study are included in the article/Supplementary material, further inquiries can be directed to the corresponding author.
