# Supplementary material for: Association between anatomical subtypes of medullary infarction and clinical outcome: a multicenter cohort study
Source: Front Neurol. 2026 Mar 24;17:1770888. doi: 10.3389/fneur.2026.1770888 (PMC13053248; doi:10.3389/fneur.2026.1770888)

SUPPLEMENTAL MATERIAL

Materials and Methods

Case Grouping Criteria on the Sagittal Plane

Based on the infarct location in the sagittal plane, patients were divided into six groups[9, 12], as detailed below: (1) Upper part: the upper medullary region, formed by the significant enlargement of the restiform body, was primarily located between the pontomedullary sulcus and the level of the inferior cerebellar peduncle. (2) Middle part: the middle medullary region, formed by the enlargement of the inferior olivary nucleus, was primarily located between the level of the inferior cerebellar peduncle and the inferior olivary nucleus. (3) Lower part: the lower medullary region, characterized by the absence of lateral enlargement and a relatively rounded shape, was primarily located between the level of the inferior olivary nucleus and the foramen magnum. (4) Upper-middle part: infarcts involving both the upper and middle regions. (5) Middle-lower part: infarcts involving both the middle and lower regions. (6) Upper-middle-lower part: infarcts involving the upper, middle, and lower regions.

Reference Category Selection

For the transverse classification, the dorsomedial subtype was selected as the reference category based on the following considerations:

(1) It had the largest sample size among medial medullary infarction subtypes (n=40), providing stable estimates;

(2) It exhibited the highest proportion of poor prognosis (65%), serving as a clinically relevant reference for comparison;

(3) It involved the most extensive anatomical region, allowing for hierarchical comparison with other subtypes.

For the TOAST classification, small-vessel occlusion was selected as the reference because:

(1) It is generally associated with a more favorable prognosis and serves as a clinically meaningful baseline for comparison with other etiologies;

(2) It represents the standard pathological mechanism of ischemic stroke;

(3) Previous studies have commonly used small-artery occlusion (SAO) as the reference category in similar analyses.

Results

Univariable Logistic Regression Analysis of 90-day mRS Prognosis

Univariable logistic regression analysis was performed on 58 variables. The results demonstrated significant associations between multiple clinical characteristics and 3-month favorable outcome (modified Rankin Scale [mRS] score ≤2) in patients with medullary infarction **(Table S4)**.

Sensitivity Analysis

In the multicenter-adjusted sensitivity analysis, hospital dummy variables were incorporated into the primary model (with Center 1 as the reference) to examine the robustness of the main findings after controlling for center effects. A total of 320 complete cases were included **(Table S5).**

Regarding the forced-entry variables, the independent predictive effects of age and admission NIHSS remained robust after adjusting for center effects. For each one-year increase in age, the probability of a favorable outcome decreased by approximately 4% (OR = 0.962, 95% CI: 0.938 - 0.987, $P = 0.003$). For each one-point increase in admission NIHSS, the probability of a favorable outcome decreased by approximately 42% (OR = 0.582, 95% CI: 0.465 - 0.729, $P < 0.001$). The effect sizes were highly consistent with the primary model, indicating that the predictive value of baseline neurological indicators was not influenced by center effects.

For treatment modality and early neurological deterioration, the independent effect of treatment modality remained statistically significant in the multicenter-adjusted model (OR = 0.305, 95% CI: 0.111 - 0.840, $P = 0.022$), with an effect size essentially identical to that of the primary model (OR = 0.331). This suggests that the negative predictive effect of treatment modality was not driven by inter-center differences. Early neurological deterioration likewise failed to reach statistical significance in this model (OR = 0.345, 95% CI: 0.101 - 1.178, $P = 0.089$), consistent with the primary model.

Regarding clinical symptoms, the independent negative predictive effect of dysphagia remained significant after multicenter adjustment (OR = 0.456, 95% CI: 0.236 - 0.884, P = 0.020), showing high concordance with the primary model results. This further confirms that impaired swallowing function is a prognostic predictor independent of center effects. Dysarthria, facial palsy, and vertigo did not achieve statistical significance (all $P > 0.05$).

For limb motor impairment subtypes, contralateral limb involvement did not reach statistical significance in this model (OR = 2.307, 95% CI: 0.876 - 6.077, P = 0.091), demonstrating attenuation compared with the primary model (P = 0.024). This suggests that the observed effect may be modestly influenced by inter-center differences in patient composition, and the results should be interpreted with caution. None of the other subtypes achieved statistical significance (all $P > 0.05$).

Regarding risk factors and laboratory indicators, coronary heart disease, hemoglobin, albumin, fasting blood glucose, and low-density lipoprotein cholesterol were not statistically significant in this model (all $P > 0.05$), which was fully consistent with the conclusions of the primary model.

For axial classification subtypes, using MMI dorsomedial as the reference, none of the subtypes reached statistical significance after adjusting for center effects (all $P > 0.05$). The direction of effects remained generally consistent with the primary model, further suggesting that the independent prognostic predictive value of axial classification is limited after controlling for baseline neurological function and center effects.

Regarding TOAST classification, large-artery atherosclerosis did not achieve statistical significance in the multicenter-adjusted model (OR = 0.669, 95% CI: 0.297 - 1.510, P = 0.334), showing marked attenuation compared with the primary model (P = 0.024). This indicates that the observed effect may be partially influenced by inter-center differences in TOAST distribution, and its independent predictive value should be interpreted cautiously in the context of center-specific distribution characteristics.

Regarding center effects, Center 2 (West China Hospital) demonstrated a significantly lower probability of favorable outcome compared with the reference Center 1 (First Affiliated Hospital of Kunming Medical University) (OR = 0.331, 95% CI: 0.153 - 0.715,

$P = 0.005$), suggesting substantial prognostic differences between centers. This may be related to variations in patient composition, treatment protocols, and rehabilitation resource allocation across centers. Center 3 (Taiyuan Hospital, Peking University First Hospital) showed no statistically significant difference compared with the reference center (OR = 1.103, $P = 0.860$).

In summary, the multicenter-adjusted sensitivity analysis demonstrated that the independent predictive effects of age, admission NIHSS, treatment modality, and dysphagia remained stable after controlling for center effects, indicating robust cross-center validity of the primary model's core conclusions. The attenuation of the large-artery atherosclerosis effect suggests that center effects warrant further investigation in etiological subtype analysis, while the significantly lower favorable outcome rate at Center 2 underscores the importance of standardized treatment protocols in multicenter studies.

In the small-subtype-excluded sensitivity analysis, the LMI oblique lateral (n = 18) and MMI medial (n = 22) subtypes with the smallest sample sizes were excluded, and 286 complete cases were included to examine the robustness of the primary model results after removing low-statistical-power subtypes **(Table S6)**.

Regarding the forced-entry variables, the independent predictive effects of age and admission NIHSS remained highly robust after excluding small subtypes. For each one-year increase in age, the probability of a favorable outcome decreased by approximately 3% (OR = 0.966, 95% CI: 0.942 - 0.991, $P = 0.007$). For each one-point increase in admission NIHSS, the probability of a favorable outcome decreased by approximately 39% (OR = 0.613, 95% CI: 0.489 - 0.768, $P < 0.001$). The effect sizes were entirely consistent with the primary model and the multicenter sensitivity analysis, further confirming the robustness of these two core predictors.

For treatment modality and early neurological deterioration, treatment modality remained statistically significant in this model (OR = 0.329, 95% CI: 0.116 - 0.930, $P = 0.036$), with effect sizes highly consistent with the primary model (OR = 0.331) and sensitivity analysis 1 (OR = 0.305). The conclusions across all three models were completely concordant, indicating robust cross-model stability of the negative predictive effect of treatment modality on prognosis. Early neurological deterioration also failed to reach statistical

significance (OR = 0.416, 95% CI: 0.129 - 1.343, *P* = 0.143), consistent with the previous two models.

Regarding clinical symptoms, the independent negative predictive effect of dysphagia remained significant after excluding small subtypes (OR = 0.468, 95% CI: 0.240 - 0.912, *P* = 0.026). The effect size was almost identical to that of the primary model (OR = 0.458) and sensitivity analysis 1 (OR = 0.456), showing high concordance across all three models and providing the strongest evidence for dysphagia as an independent prognostic predictor. Vertigo showed a *P* -value of 0.068 in this model, approaching the significance threshold and improved compared with the primary model, suggesting that this effect was slightly enhanced after adjusting the sample composition, though still not reaching statistical significance.

For limb motor impairment subtypes, contralateral limb involvement (vs. none) regained statistical significance in this model (OR = 2.952, 95% CI: 1.114 - 7.823, *P* = 0.030), with a more stable effect size compared with the primary model (OR = 2.894, *P* = 0.024) and sensitivity analysis 1 (OR = 2.307, *P* = 0.091). This suggests that after removing the interference of small subtypes, the independent predictive effect of contralateral limb involvement was further confirmed. None of the other limb subtypes reached significance (all $P > 0.05$).

Regarding risk factors and laboratory indicators, coronary heart disease and all laboratory indicators failed to reach statistical significance in this model (all $p > 0.05$), fully consistent with the conclusions of the previous two models.

For axial classification subtypes, after excluding the LMI oblique lateral and MMI medial small subtypes and using MMI dorsomedial as the reference, none of the subtypes—including LMI dorsal (OR = 1.078), LMI superficial lateral (OR = 0.590), LMI dorsolateral (OR = 0.966), and MMI ventromedial (OR = 0.406)—reached statistical significance (all $P > 0.05$). These results were consistent with the primary model conclusions, further demonstrating that the independent prognostic predictive value of axial classification remains limited after adequate adjustment for confounding factors.

Regarding TOAST classification, large-artery atherosclerosis compared with small-artery occlusion remained statistically significant in this model (OR = 0.439, 95% CI: 0.215 - 0.898, $P = 0.024$), with effect size highly consistent with the primary model (OR = 0.449, $P = 0.024$). While this effect attenuated in sensitivity analysis 1 due to center-effect adjustment, the combined results across all three models indicate that the independent negative predictive effect of large-artery atherosclerosis is robust in models without center adjustment, suggesting that inter-center TOAST distribution differences are an important factor affecting the stability of this effect.

In summary, the small-subtype-excluded sensitivity analysis demonstrated that the independent predictive effects of age, admission NIHSS, treatment modality, dysphagia, and contralateral limb motor impairment remained stable after excluding small subtypes, showing high concordance with the primary model core conclusions and further validating the reliability of the primary model results. The high concordance of effect sizes across the three models provides ample robustness evidence for the statistical inferences of this study.

In this supplementary analysis (rare-subtype-merged model), the LMI oblique lateral and MMI medial types were combined into a single "rare subtype" category. A total of 320 complete cases were included to assess the impact of subtype-merging strategies on the primary model conclusions **(Table S7)**.

Regarding the forced-entry variables, the independent predictive effects of age and admission NIHSS remained robust after merging rare subtypes. For each one-year increase in age, the probability of favorable outcome decreased by approximately 3% (OR = 0.968, 95% CI: 0.945 - 0.991, $P = 0.007$). For each one-point increase in admission NIHSS, the probability of favorable outcome decreased by approximately 41% (OR = 0.590, 95% CI: 0.475 - 0.733, $P < 0.001$). The effect sizes were highly consistent with the primary model, the multicenter-adjusted sensitivity analysis, and the small-subtype-excluded sensitivity analysis, with all four models demonstrating complete concordance in conclusions for forced-entry variables.

For treatment modality and early neurological deterioration, treatment modality remained statistically significant in the merged rare subtype model (OR = 0.309, 95% CI: 0.114 - 0.834, $P = 0.020$), with effect sizes highly consistent with the previous three models (OR

= 0.331, 0.305, and 0.329, respectively). This further supports the cross-model robustness of the negative predictive effect of treatment modality. Early neurological deterioration again did not reach statistical significance (OR = 0.411, 95% CI: 0.131 - 1.285, $P = 0.126$), with all four models yielding identical conclusions.

Regarding clinical symptoms, the independent negative predictive effect of dysphagia remained significant in this model (OR = 0.498, 95% CI: 0.263 - 0.942, $P = 0.032$). Although the effect size showed slight attenuation compared with the previous three models, statistical significance was maintained, further confirming the stability of dysphagia as an independent prognostic predictor under different subtype-grouping strategies. Dysarthria, facial palsy, and vertigo were not statistically significant (all $P > 0.05$).

For limb motor impairment subtypes, contralateral limb involvement (vs. none) maintained statistical significance in this model (OR = 2.492, 95% CI: 1.012 - 6.141, $P = 0.047$), consistent with the primary model ($P = 0.024$) and sensitivity analysis 2 ($P = 0.030$). The mildly attenuated effect observed in sensitivity analysis 1 ($P = 0.091$) was restored in this model. Across all four models, the independent predictive effect of contralateral limb involvement demonstrates overall robustness. None of the other limb subtypes reached statistical significance (all $P > 0.05$).

For axial classification subtypes, using the MMI dorsomedial subtype as the reference, none of the subtypes—including LMI dorsal (OR = 0.982), LMI superficial lateral (OR = 0.526), LMI dorsolateral (OR = 0.829), MMI ventromedial (OR = 0.417), and the merged rare subtype (OR = 1.228)—demonstrated statistical significance (all $P > 0.05$). Following the integration of the two small subtypes using the merging strategy, the rare subtype group exhibited an OR of 1.228 (95% CI: 0.311 - 4.846, $P = 0.769$), indicating no significant difference from the reference MMI dorsomedial subtype. This suggests that the prognosis of the merged subtype is comparable to that of the MMI dorsomedial subtype, further supporting the conclusion that axial classification has limited independent predictive value within the multivariable framework.

Concerning the TOAST classification, the independent negative effect of large-artery atherosclerosis compared with small-artery occlusion remained statistically significant in this model (OR = 0.471, 95% CI: 0.237 - 0.937, P = 0.032), with an effect size highly consistent with the primary model (OR = 0.449) and sensitivity analysis 2 (OR = 0.439). This further confirms the robust prognostic predictive value of the TOAST classification in models without center adjustment.

In a comprehensive comparison across all four models, age, admission NIHSS score, treatment modality, dysphagia, and contralateral limb motor impairment maintained consistent directions and similar effect sizes in all models, representing the most robust combination of independent prognostic predictors in this study. The effect of large-artery atherosclerosis was significant in the three models without center adjustment, suggesting that its independent predictive value is influenced by center effects. The independent prognostic predictive value of axial classification was not supported in any of the four models, indicating that the impact of subtype differences on prognosis is primarily mediated through baseline characteristics such as neurological deficit severity rather than direct independent effects of the subtypes themselves.

Comparison of Subtype Coefficients Across Three Models

Overall, none of the subtypes achieved statistical significance in any of the three models (all $P > 0.05$) **(Table S8)**. This conclusion remained consistent across all three model specifications, suggesting that prognostic differences among axial subtypes relative to the MMI dorsomedial type did not demonstrate independent statistical effects after adequate adjustment for baseline confounding factors.

Regarding effect direction, the LMI oblique lateral subtype exhibited odds ratio (OR) values greater than 1 across all three models (3.963 in the primary model, 3.127 in sensitivity analysis 1; excluded in sensitivity analysis 2), consistently indicating a positive trend toward a favorable prognosis compared with the MMI dorsomedial subtype. However, with only 18 cases, the confidence intervals were extremely wide (primary model: 0.492 - 31.932), resulting in substantial statistical uncertainty that precludes definitive conclusions. The LMI dorsal subtype displayed OR values close to 1 across all three models (1.116, 1.192, and 1.078), with a stable direction indicating a mild positive but minimal effect size and highly concordant results across models. The LMI superficial

lateral subtype demonstrated OR values less than 1 across all three models (0.588, 0.669, and 0.590), with a consistent direction suggesting mild negative effects, though none reached significance, indicating no statistically significant difference from the reference group. The LMI dorsolateral subtype showed OR values close to 1 across all three models (0.934, 1.313, and 0.966), with extremely weak effects and inconsistent directions, further supporting no substantial prognostic difference from the MMI dorsomedial subtype. The MMI ventromedial subtype exhibited OR values of approximately 0.4 - 0.6 across all three models (0.417, 0.556, and 0.406), with a consistent negative direction, though confidence intervals all crossed 1, failing to reach significance. The MMI medial subtype appeared only in the primary model and sensitivity analysis 1 (OR = 0.700 and 1.037, respectively), with inconsistent directions and minimal effect sizes; due to sample size limitations (n = 22), estimates were unstable, and this subtype was excluded in sensitivity analysis 2.

Regarding inter-model consistency, except for the LMI oblique lateral subtype, where wide confidence intervals led to substantial fluctuations in point estimates, OR values for all other subtypes demonstrated reasonable variation across the three models with generally consistent directions. This suggests good directional stability in subtype effect estimation under different model specifications, although none reached statistical significance.

In summary, the three-model comparison results demonstrate that the effect of axial classification on prognosis showed consistent non-significance within the multivariable framework. This conclusion remained stable regardless of center-effect adjustment or exclusion of small subtypes, providing robust cross-model evidence supporting the core finding that axial classification has limited independent prognostic value.

Table S1: Seven anatomical subtypes of medullary infarction

Anatomical Subtype	DWI Imaging	Clinical manifestations	Involved Structures	Vascular Supply	References
**LMI**					
**LMI Superficial**	Small patchy hyperintensity in the superficial lateral region of the caudal medulla, without involvement of the dorsal or deep medullary structures.	The typical clinical manifestations included contralateral hypoalgesia and thermoanesthesia of the limbs, ipsilateral hypoalgesia and thermoanesthesia of the face, vertigo, nystagmus, and ataxia.	The lateral portion of the inferior cerebellar peduncle, the superficial fibers of the spinothalamic tract, and the lateral portions of the spinal trigeminal nucleus and tract	Lateral branches of the posterior inferior cerebellar artery (PICA) or lateral branches of the vertebral artery (VA)	Kim J, 2003； Vuilleumier, 1995
**LMI Dorsolateral**	A large hyperintense area is observed in the dorsolateral medulla, extending extensively from the lateral to the dorsal regions.	Typical manifestations of Wallenberg syndrome included: contralateral impairment of pain and temperature sensation in the limbs, ipsilateral impairment of pain and temperature sensation on the face, vertigo, vomiting, nystagmus, ipsilateral Horner syndrome, dysphagia, oarseness, and ipsilateral cerebellar ataxia.	The inferior cerebellar peduncle, vestibular nuclei, spinal trigeminal nucleus and tract, spinothalamic tract, nucleus ambiguus, inferior salivatory nucleus, and reticular formation	The main trunk of the posterior inferior cerebellar artery (PICA) or the lateral branches of the vertebral artery (VA)	Kim J, 2003； Vuilleumier, 1995

**LMI Oblique Lateral**	Oblique band-like hyperintensity in the lateral medulla, presenting as an "oblique" or "band-shaped" distribution, extends from the dorsolateral to the anterolateral region.	Dysphagia and hoarseness (resulting from involvement of the nucleus ambiguus), contralateral impairment of pain and temperature sensation in the limbs, and ipsilateral impairment of pain and temperature sensation on the face (resulting from involvement of the spinal trigeminal nucleus and spinothalamic tract).	The nucleus ambiguus, the spinal trigeminal nucleus, the spinothalamic tract, and the medial portion of the inferior cerebellar peduncle	The medial branches of the posterior inferior cerebellar artery (PICA) or the perforating branches of the vertebral artery (VA)	Kim J, 2003； Kameda, 2004
**LMI Dorsal**	Small patchy or band-like hyperintensity was observed in the dorsal medulla, adjacent to the floor of the fourth ventricle, without involvement of the lateral or ventral structures of the medulla.	Ipsilateral impairment of deep sensation in the limbs (resulting from involvement of the gracile nucleus and cuneate nucleus), dysgeusia (resulting from involvement of the nucleus solitarius), visceral dysfunction (resulting from involvement of the dorsal nucleus of the vagus nerve), hiccups (resulting from involvement of the nucleus	The gracile nucleus, cuneate nucleus, dorsal motor nucleus of the vagus, solitary nucleus, and dorsal portion of the spinal trigeminal nucleus	The posterior spinal artery or the dorsal branches of the vertebral artery (VA)	Kim J, 1994

		solitarius or dorsal nucleus of the vagus nerve).			
**MMI**					
**MMI Ventromedial**	Small patchy hyperintensity in the ventromedial medulla, adjacent to the pyramid, without involvement of the medial lemniscus or medial longitudinal fasciculus	Contralateral central paralysis of the limbs (resulting from corticospinal tract involvement), ipsilateral lingual paralysis and deviation of the tongue upon protrusion (resulting from hypoglossal nucleus involvement).	The corticospinal tract (also known as the pyramidal tract), the hypoglossal nucleus, and the arcuate nucleus	Ventral perforating branches of the vertebral artery (VA) or medial branches of the anterior spinal artery	Kim J, 2009； Bassetti, 1997
**MMI Mediomedial**	A band-like hyperintensity extending from the ventral to intermediate regions of the medial medulla, involving the pyramid and medial lemniscus regions	Contralateral central paralysis of the limbs (resulting from involvement of the corticospinal tract), contralateral impairment of deep sensation in the limbs (resulting from involvement of the medial lemniscus), ipsilateral lingual paralysis (resulting from involvement of the hypoglossal nucleus), and dysarthria and dysphagia (resulting from	Corticospinal tract (pyramidal tract), medial lemniscus, hypoglossal nucleus, and partial arcuate nucleus	Medial perforating branches of the vertebral artery (VA) or medial branches of the anterior spinal artery	Kim J, 2009； Kim J, 1995

		involvement of the corticobulbar tract).			
**MMI Dorsomedial**	A large hyperintense area extending from the ventral to dorsal regions of the medial medulla, involving the pyramid, medial lemniscus, and medial longitudinal fasciculus	Contralateral central paralysis of the limbs (resulting from corticospinal tract involvement), contralateral impairment of deep sensation in the limbs (resulting from medial lemniscus involvement), ocular movement disorders and nystagmus (resulting from medial longitudinal fasciculus involvement), ipsilateral lingual paralysis (resulting from hypoglossal nucleus involvement), dysphagia and dysarthria (resulting from corticobulbar tract and nucleus ambiguus involvement), disturbance of consciousness (resulting from reticular formation involvement), and	Corticospinal tract (pyramidal tract), medial lemniscus, medial longitudinal fasciculus (MLF), hypoglossal nucleus, dorsal motor nucleus of the vagus, solitary nucleus, and part of the reticular formation	Medial perforating branches of the vertebral artery (VA), anterior spinal artery, or perforating branches of the basilar artery	Kim J, 2009；Bassetti, 1997；Tatu, 1996

	respiratory dysfunction (resulting from dorsal nucleus of the vagus nerve and nucleus solitarius involvement).	

LMI: Lateral medullary infarction, MMI: Medial medullary infarction.

Table S2: Modified Rankin Scale (mRS) Scores on Admission and Discharge

Variable	LMI Oblique Lateral	LMI Dorsal	LMI Superficial Lateral	LMI Dorsolateral	MMI Ventromedial	MMI Mediomedial	MMI Dorsomedial	Total	*P* Value
mRS Score on Admission									
0	0(0.00)	0(0.00)	1(1.52)	0(0.00)	0(0.00)	0(0.00)	0(0.00)	1(0.28)	
1	3(16.67)	3(5.17)	13(19.70)	9(8.57)	8(18.60)	3(13.64)	5(12.50)	44(12.50)	
2	5(27.78)	16(27.59)	13(19.70)	14(13.33)	8(18.60)	7(31.82)	6(15.00)	69(19.60)	$P<0.001^*$
3	8(44.44)	22(37.93)	21(31.82)	25(23.81)	10(23.26)	3(13.64)	8(20.00)	97(27.56)	
4	2(11.11)	16(27.59)	18(27.27)	54(51.43)	13(30.23)	8(36.36)	9(22.50)	120(34.09)	
5	0(0.00)	1(1.72)	0(0.00)	3(2.86)	4(9.30)	1(4.55)	12(30.00)	21(5.97)	
Total	18	58	66	105	43	22	40	352	
mRS Score on Discharge									
0	0(0.00)	0(0.00)	2(3.03)	1(0.95)	2(4.65)	0(0.00)	0(0.00)	5(1.42)	
1	4(22.22)	12(20.69)	15(22.73)	13(12.38)	9(20.93)	5(22.73)	4(10.00)	62(17.61)	
2	8(44.44)	22(37.93)	21(31.82)	21(20.00)	9(20.93)	6(27.27)	9(22.50)	96(27.27)	
3	4(22.22)	15(25.86)	20(30.30)	32(30.48)	11(25.58)	5(22.73)	7(17.50)	94(26.70)	$P<0.001^*$
4	1(5.56)	9(15.52)	6(9.09)	35(33.33)	9(20.93)	6(27.27)	8(20.00)	74(21.02)	
5	0(0.00)	0(0.00)	1(1.52)	3(2.86)	2(4.65)	0(0.00)	12(30.00)	18(5.11)	
6	1(5.56)	0(0.00)	1(1.52)	0(0.00)	1(2.33)	0(0.00)	0(0.00)	3(0.85)	
Total	18	58	66	105	43	22	40	352	

*Indicates $P<0.05$, LMI: Lateral medullary infarction, MMI: Medial medullary infarction, mRS: Modified Rankin Scale.

Table S3: Assessment of Early Neurological Deterioration

Subtype	Total	Early Deterioration n (%)	Wilson 95%CI	Admission NIHSS Score		3-Month mRS Score		Rate of good prognosis	
				Deterioration Group	Non-Deterioration Group	Deterioratio n Group	Non-Deterioration Group	Deterioratio n Group	Non-Deterioration Group
LMI oblique lateral	18	1 (5.6%)	1.0%–25.8%	5	2	6	1	0.0%	88.2%
LMI dorsal	58	2 (3.4%)	1.0%–11.7%	3.5	2	2	2	100.0%	69.6%
LMI superficial lateral	66	3 (4.5%)	1.6%–12.5%	2	2	6	2	33.3%	66.7%
LMI dorsolateral	105	13 (12.4%)	7.4%–20.0%	4	3	3	2	23.1%	58.7%
MMI ventromedial	43	5 (11.6%)	5.1%–24.5%	9	2	4	2	20.0%	60.5%
MMI mediomedial	22	1 (4.5%)	0.8%–21.8%	6	3	3	2	0.0%	61.9%
MMI dorsomedial	40	10 (25.0%)	14.2%–40.2%	11	5	5	3	0.0%	46.7%
Overall	352	35 (9.9%)	7.2%–13.5%	5	3	4	2	20.0%	63.1%

Early neurological deterioration was defined as an increase in NIHSS score ≥4 points or death during hospitalization. Good prognosis was defined as 3-month mRS ≤2. Incidence rates are presented with Wilson 95% confidence intervals. LMI: Lateral medullary infarction, MMI: Medial medullary infarction.

Table S4: Univariable Logistic Regression

Variable	β Coefficient	SE	OR (95%CI)	*P*-value
**Age**	-0.0214	0.0076	0.979 (0.964–0.994)	0.005*
**Male sex**	0.2144	0.2751	1.239 (0.723–2.124)	0.436
**Admission NIHSS**	-0.4565	0.0682	0.634 (0.554–0.724)	<0.001*
**Treatment modality**	-1.148	0.4038	0.317 (0.144–0.700)	0.004*
**END**	-1.9224	0.4383	0.146 (0.062–0.345)	<0.001*
**Clinical Symptoms**				
Sensory disturbance	-0.0253	0.0478	0.975 (0.888–1.071)	0.597
Limb motor impairment (vs. none)	-0.3868	0.1041	0.679 (0.554–0.833)	<0.001*
Ipsilateral	-0.0365	0.2653	0.964 (0.573–1.622)	0.891
Contralateral	-0.2937	0.2406	0.746 (0.465–1.195)	0.222
Quadriplegia	-2.2657	0.6338	0.104 (0.030–0.359)	<0.001*
Other	0.1584	0.7385	1.172 (0.276–4.982)	0.830
Dysarthria	-1.2447	0.227	0.288 (0.185–0.449)	<0.001*
Dysphagia	-0.7954	0.2267	0.451 (0.289–0.704)	<0.001*
Facial palsy	-0.7359	0.2277	0.479 (0.307–0.749)	0.001*
Ataxia	-0.3312	0.2177	0.718 (0.469–1.100)	0.128
Dizziness	0.1598	0.2228	1.173 (0.758–1.816)	0.473
Vertigo	0.6898	0.2373	1.993 (1.252–3.174)	0.004*
Headache	0.1503	0.241	1.162 (0.725–1.864)	0.533
Diplopia	0.0825	0.374	1.086 (0.522–2.260)	0.825
Nystagmus	-0.3029	0.2686	0.739 (0.436–1.250)	0.259
Vomiting	0.2401	0.2206	1.271 (0.825–1.959)	0.276
Hiccups	-0.4002	0.3443	0.670 (0.341–1.316)	0.245
Ocular motor impairment	-0.4702	0.4159	0.625 (0.277–1.412)	0.258
Pathological signs	-0.4252	0.2593	0.654 (0.393–1.087)	0.101
**Risk Factors**				
Hypertension	0.0115	0.2303	1.012 (0.644–1.589)	0.960
Diabetes mellitus	-0.3182	0.227	0.727 (0.466–1.135)	0.161
Hyperlipidemia	0.2156	0.2503	1.241 (0.760–2.026)	0.389
Hyperhomocysteinemia	0.2054	0.253	1.228 (0.748–2.016)	0.417
Atrial fibrillation	0.3428	0.8729	1.409 (0.255–7.796)	0.695
Coronary heart disease	-0.7207	0.4128	0.486 (0.217–1.092)	0.081
History of myocardial infarction	0.0502	0.9194	1.051 (0.173–6.373)	0.956
Cardiac insufficiency	-0.5947	0.6798	0.552 (0.146–2.091)	0.382

History of TIA	-0.3581	1.4184	0.699 (0.043–11.267)	0.801
Alcohol consumption	-0.2299	0.2255	0.795 (0.511–1.236)	0.308
Smoking	-0.0732	0.2167	0.929 (0.608–1.421)	0.735
**Laboratory Parameters**				
Hemoglobin	0.016	0.0056	1.016 (1.005–1.027)	0.004*
Platelet count	0.0005	0.0012	1.000 (0.998–1.003)	0.700
APTT	0.0103	0.0167	1.010 (0.978–1.044)	0.536
PT	0.0158	0.0265	1.016 (0.965–1.070)	0.551
INR	-1.0177	0.9402	0.361 (0.057–2.282)	0.279
Blood urea nitrogen	-0.0715	0.0526	0.931 (0.840–1.032)	0.174
Albumin	0.0442	0.0261	1.045 (0.993–1.100)	0.090
Creatinine	0.0045	0.0047	1.005 (0.995–1.014)	0.336
Fasting blood glucose	-0.0644	0.0357	0.938 (0.874–1.006)	0.071
ALT	-0.0007	0.0038	0.999 (0.992–1.007)	0.851
AST	-0.0032	0.0043	0.997 (0.988–1.005)	0.460
Triglycerides	0.0609	0.0897	1.063 (0.892–1.267)	0.497
Total cholesterol	0.0969	0.0859	1.102 (0.931–1.304)	0.259
HDL cholesterol	-0.4307	0.4167	0.650 (0.287–1.471)	0.301
LDL cholesterol	0.1794	0.108	1.196 (0.968–1.479)	0.097
**Axial Classification (vs. MMI dorsomedial)**				
LMI oblique lateral	1.3078	0.6421	3.698 (1.051–13.016)	0.042*
LMI dorsal	0.6204	0.3115	1.860 (1.010–3.425)	0.046*
LMI superficial lateral	0.3299	0.2847	1.391 (0.796–2.430)	0.247
LMI dorsolateral	-0.2641	0.2353	0.768 (0.484–1.218)	0.262
MMI ventromedial	-0.1396	0.3282	0.870 (0.457–1.655)	0.671
MMI mediomedial	0.0125	0.4478	1.013 (0.421–2.436)	0.978
**TOAST Classification (vs. Small-artery occlusion)**				
Large-artery atherosclerosis	-0.8855	0.2271	0.412 (0.264–0.644)	<0.001*
Other	0.1636	0.3285	1.178 (0.619 – 2.242)	0.618

*Indicates *P*<0.05, APTT: Activated Partial Thromboplastin Time; ALT: Alanine Aminotransferase; AST: Aspartate Aminotransferase; INR: International Normalized Ratio; LMI: Lateral medullary infarction, MMI: Medial medullary infarction, NIHSS: National Institutes of Health Stroke Scale; PT: Prothrombin Time; SE: Status Epilepticus; TIA: Transient Ischemic Attack.

Table S5: Sensitivity Analysis – Multicenter Model

Variable	β Coefficient	SE	OR (95%CI)	*P*-value
**Age**	-0.0386	0.0128	0.962 (0.938–0.987)	0.003*
**Admission NIHSS**	-0.5411	0.1147	0.582 (0.465–0.729)	<0.001*
**Treatment modality**	-1.1864	0.5163	0.305 (0.111–0.840)	0.022*
**Early neurological deterioration**	-1.0652	0.6272	0.345 (0.101–1.178)	0.089
**Dysarthria**	-0.3495	0.3426	0.705 (0.360–1.380)	0.308
**Dysphagia**	-0.7842	0.3371	0.456 (0.236–0.884)	0.020*
**Facial palsy**	0.2467	0.3438	1.280 (0.652–2.511)	0.473
**Vertigo**	0.2817	0.3316	1.325 (0.692–2.539)	0.396
**Coronary heart disease**	-0.5024	0.5397	0.605 (0.210–1.743)	0.352
**Hemoglobin**	0.0021	0.0085	1.002 (0.986–1.019)	0.808
**Albumin**	-0.0142	0.044	0.986 (0.905–1.075)	0.746
**Fasting blood glucose**	-0.0058	0.0536	0.994 (0.895–1.104)	0.913
**Low-density lipoprotein**	0.1286	0.1446	1.137 (0.857–1.510)	0.374
**Limb motor impairment**				
Ipsilateral	0.1673	0.4064	1.182 (0.533–2.622)	0.681
Contralateral	0.8359	0.4942	2.307 (0.876–6.077)	0.091
Quadriplegia	-1.0157	0.8114	0.362 (0.074–1.776)	0.211
Other	0.2781	1.0481	1.321 (0.169–10.301)	0.791
**Transverse Subtype**				
LMI oblique lateral	1.1401	1.0481	3.127 (0.401–24.396)	0.277
LMI dorsal	0.1756	0.7372	1.192 (0.281–5.056)	0.812
LMI superficial lateral	-0.4017	0.7092	0.669 (0.167–2.687)	0.571
LMI dorsolateral	0.2725	0.6555	1.313 (0.363–4.746)	0.678
MMI ventromedial	-0.5864	0.7289	0.556 (0.133–2.322)	0.421
MMI medial	0.0364	0.807	1.037 (0.213–5.044)	0.964
**TOAST Classification**				
Large-artery atherosclerosis	-0.4013	0.415	0.669 (0.297–1.510)	0.334
Other	-0.0521	0.5439	0.949 (0.327–2.757)	0.924
**Center: 2**	-1.1047	0.3926	0.331 (0.153–0.715)	0.005*
**Center: 3**	0.098	0.5558	1.103 (0.371–3.278)	0.860

*Indicates $P<0.05$, hospital dummy variables were included (reference: Center 1). $n = 320$, AIC = 354.73, $R^2 = 0.3145$, Center 2: West China Hospital of Sichuan University, Center 3: Peking University First Hospital Taiyuan Hospital. LMI: Lateral medullary infarction, MMI: Medial medullary infarction

Table S6: Sensitivity Analysis – Excluding Small Subtypes

Variable	β Coefficient	SE	OR	OR (95%CI)	*P*-value
**Age**	-0.0348	0.013	0.9658	0.966 (0.942–0.991)	0.007*
**Admission NIHSS**	-0.4895	0.1153	0.6129	0.613 (0.489–0.768)	<0.001*
**Treatment modality**	-1.1129	0.531	0.3286	0.329 (0.116–0.930)	0.036*
**Early neurological deterioration**	-0.8767	0.5979	0.4162	0.416 (0.129–1.343)	0.143
**Dysarthria**	-0.3693	0.3541	0.6913	0.691 (0.345–1.384)	0.297
**Dysphagia**	-0.7588	0.3403	0.4682	0.468 (0.240–0.912)	0.026*
**Facial palsy**	0.0613	0.3467	1.0633	1.063 (0.539–2.098)	0.860
**Vertigo**	0.6131	0.3357	1.8462	1.846 (0.956–3.565)	0.068
**Coronary heart disease**	-0.5405	0.5936	0.5825	0.582 (0.182–1.864)	0.362
**Hemoglobin**	0.0067	0.0081	1.0067	1.007 (0.991–1.023)	0.409
**Albumin**	-0.0534	0.044	0.948	0.948 (0.870–1.033)	0.224
**Fasting blood glucose**	0.031	0.0526	1.0315	1.031 (0.930–1.144)	0.556
**Low-density lipoprotein**	0.0215	0.0918	1.0217	1.022 (0.853–1.223)	0.815
**Limb motor impairment subtype**					
Ipsilateral	0.3562	0.3984	1.4279	1.428 (0.654–3.118)	0.371
Contralateral	1.0824	0.4973	2.9517	2.952 (1.114–7.823)	0.030*
Quadriplegia	-1.4266	0.9289	0.2401	0.240 (0.039–1.483)	0.125
Other	1.4045	1.2092	4.0734	4.073 (0.381–43.576)	0.245
**Transverse Subtype**					
LMI dorsal	0.0753	0.7384	1.0782	1.078 (0.254–4.584)	0.919
LMI superficial lateral	-0.528	0.7109	0.5898	0.590 (0.146–2.376)	0.458
LMI dorsolateral	-0.0351	0.6523	0.9655	0.966 (0.269–3.468)	0.957
MMI ventromedial	-0.9013	0.7119	0.406	0.406 (0.101–1.639)	0.205
**TOAST Classification**					
Large-artery atherosclerosis	-0.8233	0.3651	0.439	0.439 (0.215–0.898)	0.024*
Other	-0.3464	0.5232	0.7072	0.707 (0.254–1.972)	0.508

*Indicates $P<0.05$, LMI oblique lateral (n = 18) and MMI medial (n = 22) were excluded. n = 286, AIC = 325.00, R^2 = 0.2932.

Table S7: Sensitivity Analysis – Merging Rare Subtypes

Variable	β Coefficient	SE	OR	OR (95%CI)	*P*-value
Age	-0.033	0.0121	0.9676	0.968 (0.945–0.991)	0.007*
Admission NIHSS	-0.528	0.1108	0.5898	0.590 (0.475–0.733)	<0.001*
Treatment modality	-1.1746	0.5066	0.3089	0.309 (0.114–0.834)	0.020*
Early neurological deterioration	-0.8892	0.5816	0.411	0.411 (0.131–1.285)	0.126
Dysarthria	-0.3293	0.3341	0.7194	0.719 (0.374–1.385)	0.324
Dysphagia	-0.6973	0.325	0.4979	0.498 (0.263–0.942)	0.032*
Facial palsy	0.1142	0.3262	1.1209	1.121 (0.591–2.125)	0.726
Vertigo	0.4029	0.3182	1.4961	1.496 (0.802–2.791)	0.205
Coronary heart disease	-0.454	0.5224	0.6351	0.635 (0.228–1.768)	0.385
Hemoglobin	0.0076	0.0079	1.0076	1.008 (0.992–1.023)	0.336
Albumin	-0.036	0.0421	0.9646	0.965 (0.888–1.048)	0.392
Fasting blood glucose	-0.0386	0.0482	0.9621	0.962 (0.875–1.057)	0.423
Low-density lipoprotein	0.0978	0.1421	1.1028	1.103 (0.835–1.457)	0.491
**Transverse Subtype**					
Ipsilateral	0.4512	0.3943	1.5701	1.570 (0.725–3.401)	0.253
Contralateral	0.9133	0.4601	2.4925	2.492 (1.012–6.141)	0.047*
Quadriplegia	-1.0134	0.8169	0.363	0.363 (0.073–1.800)	0.215
Other	0.7327	1.0039	2.0808	2.081 (0.291–14.885)	0.465
**Transverse Subtype**					
LMI dorsal	-0.0186	0.7097	0.9815	0.982 (0.244–3.945)	0.979
LMI superficial lateral	-0.6433	0.6863	0.5256	0.526 (0.137–2.017)	0.349
LMI dorsolateral	-0.1879	0.6271	0.8287	0.829 (0.242–2.833)	0.764
MMI ventromedial	-0.8735	0.7062	0.4175	0.417 (0.105–1.666)	0.216
Rare subtypes (merged)	0.2055	0.7003	1.2281	1.228 (0.311–4.846)	0.769
**TOAST Classification**					
Large-artery atherosclerosis	-0.7533	0.3509	0.4708	0.471 (0.237–0.937)	0.032*
Other	-0.4683	0.4892	0.6261	0.626 (0.240–1.633)	0.339

*Indicates $P<0.05$, LMI oblique lateral and MMI medial were merged as "Rare subtypes." n = 320, AIC = 360.20, R^2 = 0.2881. LMI: Lateral medullary infarction, MMI: Medial medullary infarction.

Table S8: Comparison of Subtype Coefficients Across Three Models

Axial Subtype (vs. MMI dorsomedial)	Primary Model 95%CI	Primary Model *P*-value	Sensitivity: Center 95%CI	Sensitivity: Center *P*-value	Sensitivity: Small Subtype 95%CI	Sensitivity: Small Subtype *P*-value
LMI oblique lateral	3.963 (0.492–31.932)	0.196	3.127 (0.401–24.396)	0.277		
LMI dorsal	1.116 (0.274–4.539)	0.878	1.192 (0.281–5.056)	0.812	1.078 (0.254–4.584)	0.919
LMI superficial lateral	0.588 (0.152–2.281)	0.443	0.669 (0.167–2.687)	0.571	0.590 (0.146–2.376)	0.458
LMI dorsolateral	0.934 (0.271–3.220)	0.914	1.313 (0.363–4.746)	0.678	0.966 (0.269–3.468)	0.957
MMI ventromedial	0.417 (0.105–1.662)	0.215	0.556 (0.133–2.322)	0.421	0.406 (0.101–1.639)	0.205
MMI medial	0.700 (0.155–3.172)	0.644	1.037 (0.213–5.044)	0.964		

LMI: Lateral medullary infarction, MMI: Medial medullary infarction.

Figure S1: Bar Chart of Poor Prognosis by Cross-Classification of Transverse and Sagittal Subtypes.

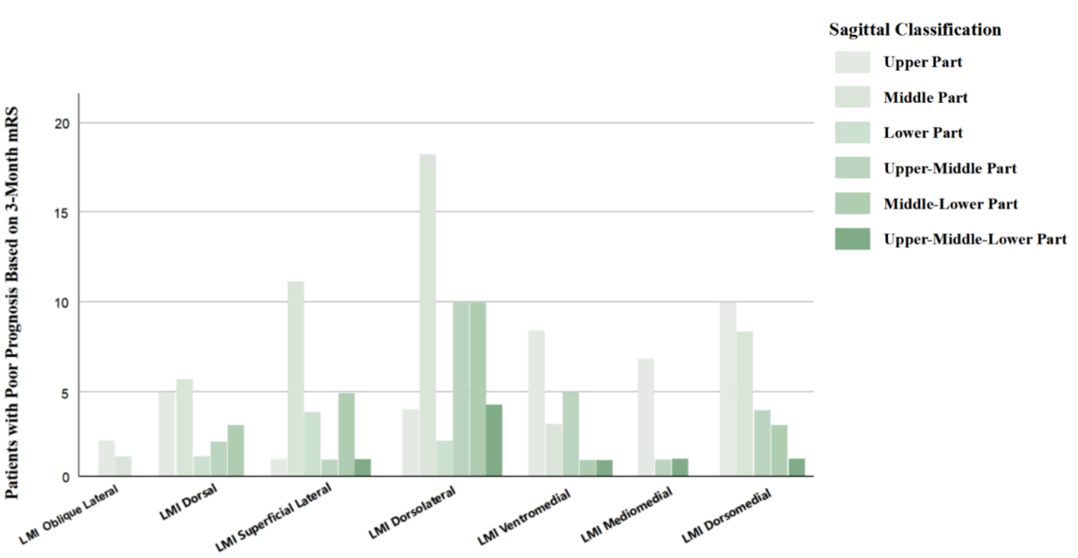

Figure S2: 90-day Prognostic Outcomes Across Different Transverse Classifications

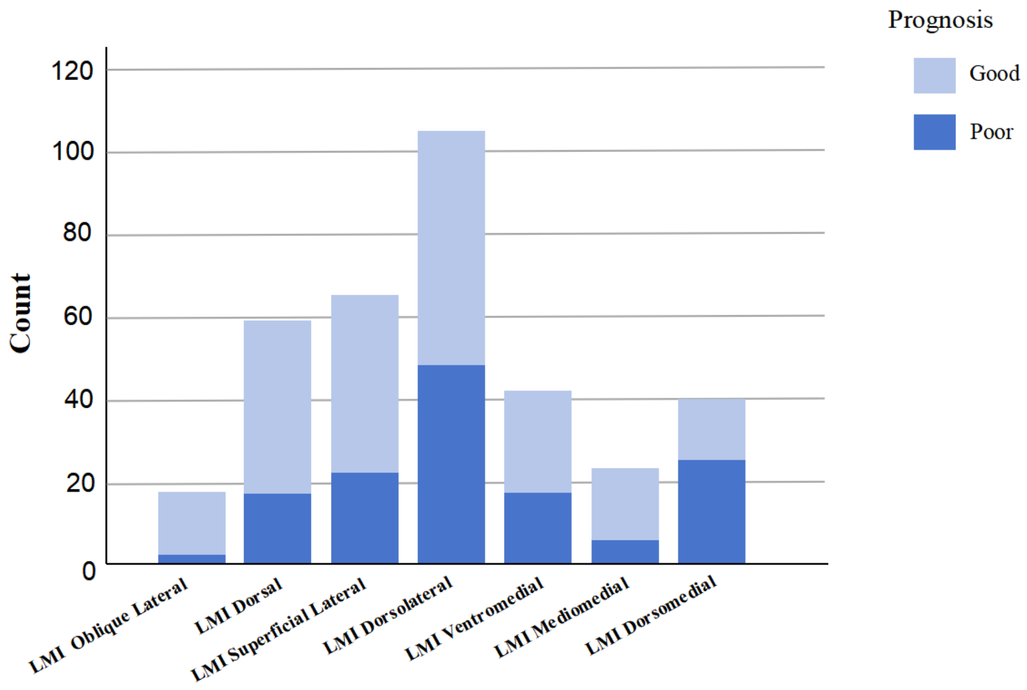

Figure S3: Incidence of Anatomical Subtypes in Medullary Infarction

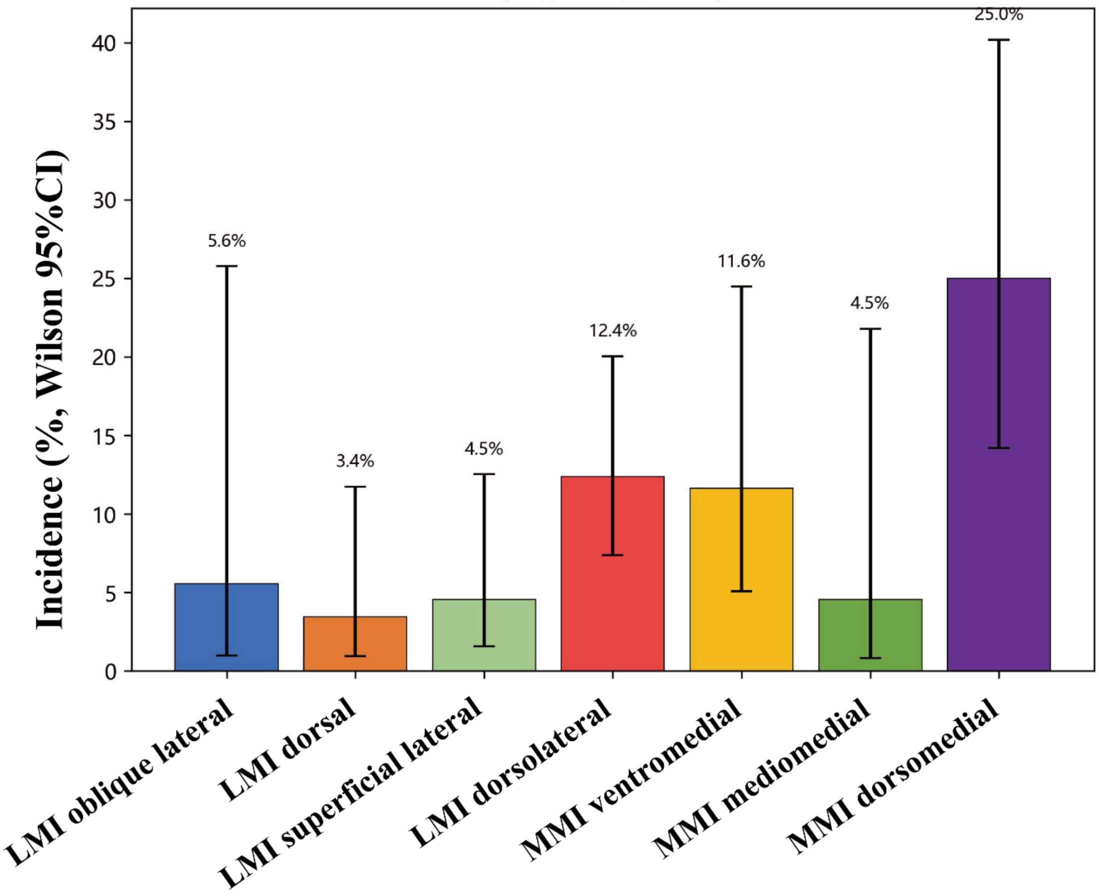

Supplement: Supplementary file 1 [file Data_Sheet_1.PDF]
